# Origin and Evolution of TRIM Proteins: New Insights from the Complete TRIM Repertoire of Zebrafish and Pufferfish

**DOI:** 10.1371/journal.pone.0022022

**Published:** 2011-07-15

**Authors:** Pierre Boudinot, Lieke M. van der Aa, Luc Jouneau, Louis Du Pasquier, Pierre Pontarotti, Valérie Briolat, Abdenour Benmansour, Jean-Pierre Levraud

**Affiliations:** 1 Virologie et Immunologie Moléculaires, Institut National de la Recherche Agronomique, Jouy-en-Josas, France; 2 Cell Biology and Immunology Group, Wageningen University, Wageningen, The Netherlands; 3 Institute of Zoology and Evolutionary Biology, University of Basel, Basel, Switzerland; 4 Equipe Evolution Biologique et Modélisation UMR 6632 Université de Aix Marseille I/CNRS, Centre St Charles, Marseille, France; 5 Unité Macrophages et Développement de l'Immunité, Institut Pasteur, Paris, France; 6 URA 2578 du Centre National de la Recherche Scientifique, Paris, France; California State University Fullerton, United States of America

## Abstract

Tripartite motif proteins (TRIM) constitute a large family of proteins containing a RING-Bbox-Coiled Coil motif followed by different C-terminal domains. Involved in ubiquitination, TRIM proteins participate in many cellular processes including antiviral immunity. The TRIM family is ancient and has been greatly diversified in vertebrates and especially in fish. We analyzed the complete sets of *trim* genes of the large zebrafish genome and of the compact pufferfish genome. Both contain three large multigene subsets - adding the *hsl5*/*trim35*-like genes (*hltr*) to the *ftr* and the *btr* that we previously described - all containing a B30.2 domain that evolved under positive selection. These subsets are conserved among teleosts. By contrast, most human *trim* genes of the other classes have only one or two orthologues in fish. Loss or gain of C-terminal exons generated proteins with different domain organizations; either by the deletion of the ancestral domain or, remarkably, by the acquisition of a new C-terminal domain. Our survey of fish *trim* genes in fish identifies subsets with different evolutionary dynamics. *trims* encoding RBCC-B30.2 proteins show the same evolutionary trends in fish and tetrapods: they evolve fast, often under positive selection, and they duplicate to create multigenic families. We could identify new combinations of domains, which epitomize how new *trim* classes appear by domain insertion or exon shuffling. Notably, we found that a cyclophilin-A domain replaces the B30.2 domain of a zebrafish *fintrim* gene, as reported in the macaque and owl monkey antiretroviral TRIM5α. Finally, *trim* genes encoding RBCC-B30.2 proteins are preferentially located in the vicinity of MHC or MHC gene paralogues, which suggests that such *trim* genes may have been part of the ancestral MHC.

## Introduction

**Figure 1:**
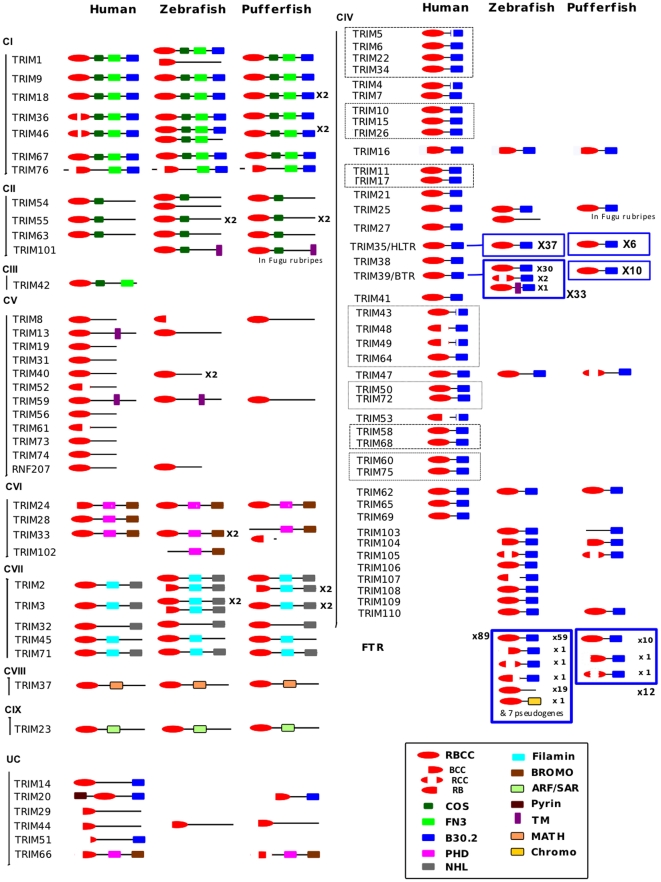
TRIM proteins from zebrafish and pufferfish. Classification of fish TRIM proteins based on their C-terminal domain(s) and the categories proposed by Short & Cox in Ref. 3. Previously unreported TRIM proteins found in fish were tentatively numbered TRIM101-111. Conserved TRIM proteins are represented on the left panel (Classes I–III and V–IX). Other TRIM proteins are shown on the right panel (Class IV). Dotted lines delimit groups of closely related human TRIM (modified from Ref. 6) corresponding to a diversification that occurred during tetrapod evolution. Blue frames indicate multigenic families observed in teleost fish. RBCC: Ring-BBox-Coiled Coil; COS: C-terminal subgroup one signature; FN3: Fibronectin, type III; B30.2: PRY/SPRY domain; PHD: Plant Homeo Domain; NHL: NCL-1, HT2A and Lin-41 repeat; Filamin: named from the protein Filamin; Bromo: acetylated lysine binding domain; ARF/SAR: from ARF and SAR GTP binding proteins; Pyrin: a member of the six-helix bundle death domain-fold superfamily; TM: transmembrane; Math: meprin and TRAF homology domain; Chromo: CHRromatin Organization Modifier domain.

The tripartite motif (TRIM) family –also known as the N-terminal RING finger/B-box/coiled coil (RBCC) family– play major roles in development, tumor suppression, disease pathology and viral restriction/sensing [1], [2]. This tripartite motif is associated with diverse C-terminal domains, which often determine the specificity of the interactions of TRIMs with other proteins. Hence, TRIM proteins associate a RING-dependent E3 ubiquitin ligase activity with the capacity to build multiprotein complexes though interactions with their CC and C-terminal domains. Human TRIM proteins have been classified into 9 architectural subsets on the basis of their C-terminal domains, subcellular compartmentalization and functionality ([3], Figure 1). The B30.2 domain [4] found in Class-I and Class-IV TRIM proteins is constituted by the juxtaposition of a PRY and a SPRY domain, and is also known as PRY/SPRY domain [5].

In a survey of the TRIM family in various species, Sardiello *et al.* distinguished two groups: the *trim* genes from group 1 contain a variety of C-terminal domains and are generally well conserved among distant species, while members of group 2 correspond to the structural Class IV subgroup which evolve much faster, display lower levels of amino acid conservation in distant species and are subjected to different selection pressures [6]. Importantly, the Class IV TRIM proteins include multiple members involved in antiviral immunity at various levels of the interferon (IFN) signalling cascade. For instance TRIM25 is required for viral RNA sensing performed by the cytoplasmic helicase RIG-I, leading to IFN production [7]. Other class IV TRIM proteins also control signalling pathways that lead to IFN production: TRIM27 represses NFκB and IRF3/IRF7 [8] while TRIM21 ubiquitylates IRF3/IRF7 and IRF8 [9], [10], [11]. On the other hand, TRIM5α - which was described as a strong restriction factor for HIV-1 in rhesus macaque - directly targets retroviruses [12]. The TRIM5α B30.2 domain binds the nucleocapsid of incoming viral particles and accelerates the uncoating of the viral core, while the RING/B-box domains are essential for the localization in specific cytoplasmic ‘bodies’ [13], [14], [15] and mediate a TRIM5α higher-order self association that increases avidity for retroviral capsids [16], [17]. The structure of the B30.2 domain is a β-sandwich core with ligand-binding loops at the top that are variable and determine the specificity of the interaction. Ligand-binding loops of the TRIM5α B30.2 domain diversified during the evolution of primates, ensuring efficient restriction of specific retroviruses in the different species [18], [19]. Thus, while TRIMs constitute an ancient family with members involved in basic cellular processes in practically all bilateria and pre-bilateria phyla [20], it seems that several subsets have been recruited and diversified for antiviral immunity during the evolution of vertebrates. However, the specific modalities of these apparently independent multiplication and diversification events are still poorly understood.

Teleost fishes offer an intriguing model for a comparative study of the TRIM family because of their ancient separation from the tetrapods, their great diversity and the considerable variation in the number of *trim* genes in their genome. In addition, the zebrafish (*Danio rerio*, Hamilton) is an important vertebrate model for developmental biology, and more recently, for host-pathogen interactions. Therefore the identification and classification of its many *trim* genes is important for these fields of biological study—and the further development of zebrafish as a model for vertebrates. Sardiello *et al.* listed 240 zebrafish *trim* genes, but without providing classification [6]. During our investigation of IFN-inducible class IV trim homologues in trout, we identified 84 *fintrim* (*ftr)* and 33 *trim39/bloodthirsty*-like (*btr*) genes [21], implying that the zebrafish genome contains at least 117 class IV genes, and probably many more. *Ftr*s do not have true orthologues in mammals, thus should have a specific function in fish defense. Apart from *ftrs* and *btrs*, the zebrafish gene database at zfin.org currently lists only 21 additional *trim* genes.

We therefore performed an exhaustive description of *trim* genes in two fish species that followed different genomic evolutionary histories – zebrafish (*Danio rerio*) and spotted green pufferfish (*Tetraodon nigroviridis*) - updating and completing the lists provided in [6]. In contrast to other *trim* genes that are generally conserved through vertebrates with conserved expression patterns, the vast majority of fish class IV *trim* genes belongs to three multigenic families of which the B30.2 domain has evolved under positive selection. Our systematic analysis of *trim* genes also led to the identification of genes that have lost, gained or replaced their C terminus domain, providing a good illustration of the mechanisms giving birth to new *trim* classes.

## Results

### TRIM classes reflect two distinct evolutionary pathways in fish

The complete repertoire of *trim* genes was determined in two fish species using combined approaches of genome scanning for protein domains and sequence comparison (see Material and Methods for details). Among fish species with available genomes, we chose the zebrafish and the spotted green pufferfish because they are phylogenetically distinct with an estimated 300 My time of divergence [22] and followed distinct genomic evolutions. They have different gene contents (15315 genes in the pufferfish compared to 23569 in the zebrafish, in Ensembl release 57) and have been subjected to different events of genome expansion/contraction. Thus, we expected that zebrafish genome would contain an expanded repertoire of *trim* genes while the compact pufferfish genome may have a “minimal” *trim* repertoire.

Zebrafish and pufferfish *trim* repertoires are presented in Figure 1, where they are compared to the human repertoire. Detailed information about the genes is provided in Supplemental Figures S1, S2 and S3. Fish *trim* genes were named after the human orthologues, following the Ensembl annotations confirmed by the analysis of the domain organization of the protein. When a close paralogue of such a fish *trim* was found with a highly similar organization but lacking the terminal domain, it was considered as another orthologue (i.e. a co-orthologue) and was therefore given the same name with a “like” suffixe. When a fish *trim* had no orthologue in human or in the mouse, we named it from available publications or we attributed a temporary “trim101-110” annotation, waiting for a definitive nomenclature.

We found 208 *trim* genes in the zebrafish (Zv9 assembly) and 66 in pufferfish (Figure 1), compared to 75 and 67 genes reported in human and in the mouse respectively [23], [24]. Sardiello *et al.* had reported 240 *trim* genes in zebrafish and 58 in pufferfish [6]; the large discrepancy observed in zebrafish was due to the fact the list established by Sardiello *et al.* was derived from a search in ESTs that were not matched to the genome sequence. This procedure resulted in frequent inclusion of the same sequence under two accession numbers, or inclusion of allelic variants. Orthologues of human *trim* genes for all classes excluding C-III (RBCC-COS-FN3) were present in both zebrafish and pufferfish. The main *trim* categories, with the possible exception of C-III, were therefore already defined in the common ancestor of fishes and tetrapods.

As shown below, *trim* genes could be separated into two main subsets reflecting their evolutionary dynamics, in complete agreement with Sardiello *et al*. [6].

One or two counterparts were found in fish for the majority of the human *trim* genes belonging to the classes I, II and VI–IX (Figure 1, left column). The presence of two co-orthologues of a given human gene in both fish species likely reflects the ancestral duplication of teleost genomes [25], [26]. The term “co-orthologue” is employed here to describe the evolutionary relationship of two or more paralogous genes with their counterpart in another species. Co-orthologue is synonymous of “inparalogue” [27]. For these *trim* classes, pufferfish and zebrafish *trim* repertoires were overall very similar, with a few exceptions: *trim40* (ClassV), *rnf207* (ClassV), *trim102* (ClassVI), as well as a *trim1-like*, a *trim54-like* and a *trim3-like* were found in zebrafish only while *trim20*, *trim66*, *trim18-2* and *trim2-like* were found only in pufferfish. These exceptions are likely due to local events of gene duplication or loss.

TRIM lacking a unique C terminal domain (Class C–V) could also be included into this subset as they never have more than two fish orthologues, even though many class V human genes lack a fish counterpart: only 6 zebrafish and 2 pufferfish counterparts were found for 12 human genes. In fact, genes closely related to members of other classes that have lost their C terminal domain - for example zebrafish *trim54like* - could fall into the class V as defined above. Thus, at least in fish, *trim* genes with no specific C-terminal domain do not constitute a homogeneous group.

**Figure 2:**
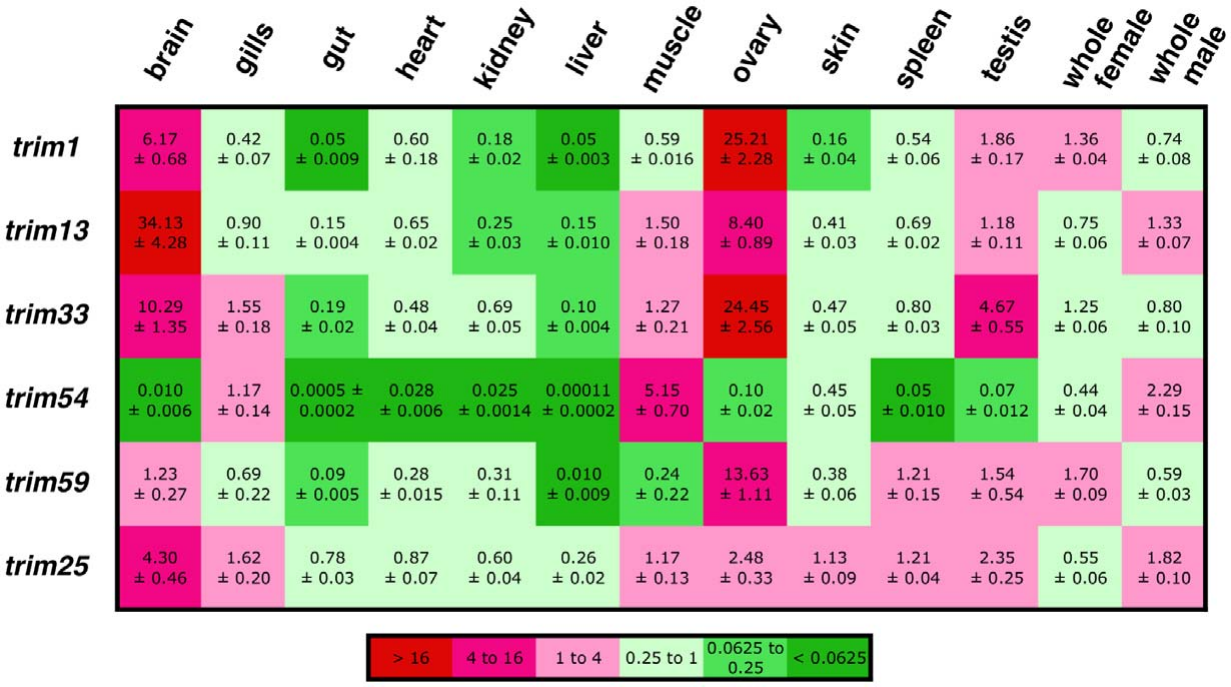
Expression profile of selected trim genes. The expression of 6 zebrafish *trim* genes was measured by quantitative RT-PCR in pools of tissues from 10–12 animals. E1f-α was used as a housekeeping gene, and the relative expression levels of *trim* genes were normalized on the geometric mean of the values measured for “whole males” and “whole females”, to take both sexes into account in the normalization. The data are represented as a heat map, with expression level and standard deviation is indicated for each condition.

The conservation of these TRIM proteins between teleosts and mammals strongly suggests that they carry out conserved functions. Such a hypothesis would be reinforced if these proteins had similar tissue-specific expression patterns. To test this hypothesis, we selected a subset of *trim* genes with a clear orthology relationship between human and zebrafish (Supplemental Figure S4) and tissue-specific expression described in mammals, and measured their expression in various organs of adult zebrafish by qRT-PCR (Figure 2). *Trim1* (also known as *FXY2 or MID2*) has been reported to be expressed “in low abundance in brain and lung, with even lower levels in heart, liver and kidney” by northern blot analysis of mouse tissues [28]. Indeed, in zebrafish, *trim1* was expressed at a higher level in brain than in heart, liver or kidney (Figure 2). In the case of *trim13* (aka *RFP2*) strongest expression was found in the testis (ovary was not tested) for both mouse and human [29]; the situation was different in the zebrafish, where only moderate levels of *trim13* were measured in the testis, although levels were high in the ovary (Figure 2). Apart from gonads, the zebrafish tissue with the highest level of expression was the brain, in agreement with mouse, but not human northern blot data [29] – note however that strong staining with anti-RFP2 antibody is detected in human brain samples (www.proteinatlas.org). Expression of *trim25* (also known as *efp*) –the function of which may suggest a rather uniform expression [7]- has been tested by RNAse protection assay in mouse tissues; abundant levels were observed in uterus, ovary and placenta, medium levels in the mammary gland, and lower levels in liver, spleen, kidney, heart, lung and thymus, and only a faint band in brain [30]. A rather ubiquitous pattern was found in zebrafish (Figure 2); a discrepancy with mouse data was the relatively high expression in brain. By Northern blot, *trim33* (or *TIF1γ*) expression was found to be highest in mouse testis, then in liver, heart, brain and kidney, weak in spleen and lung and very low in skeletal muscle [31]. Among the corresponding zebrafish organs, *trim33* expression was highest in brain, then in testis; however, it was fairly low in liver, while intermediate in muscle (Figure 2). *Trim54* (also known as *MURF*) constitutes the most clear-cut example or tissue-specific expression, with an almost exclusive expression in heart and skeletal muscle [32]. A similar pattern was observed in zebrafish: expression was strong in skeletal muscle, and extremely low in visceral organs – the low heart expression was, however, a remarkable difference (Figure 2). Finally, *trim59* (or *Mrf1*) expression data in mammals are rather conflicting; in mouse, expression was found to be strong in testis, moderate in spleen, weak in brain and heart, and very low in lung, liver, kidney or skeletal muscle [33]; while in humans, highest levels were detected in skeletal muscle, heart, liver and lung [34]. In zebrafish, strong expression was found in ovary, and low levels in gut, level or muscle (Figure 2). In conclusion, although the variety of techniques and organs sampled in the various published studies makes comparisons quite difficult, similar patterns of expression can often be observed between mammalian and fish tissues (if one excludes gonads, where extreme expression levels are frequent), likely reflecting conservation of function for these genes.

Class IV trims (RBCC-B30.2) followed a different evolutionary pathway. No obvious counterpart could be found in fish of the majority of human and mouse *trim* genes belonging to this class (Figure 1, right column). Our analysis identified fish orthologues only for *trim16*, *trim25*, *trim35*, *trim39* (the *btr* family), *trim47* and *trim62*. Reciprocally, several fish class IV genes had no counterparts in mammals, such as the *fintrims* and the *trim103-110*. Strikingly, several Class-IV members constitute multigenic subsets in fish. Two of these multigene subsets possess a unique human counterpart *trim39* and *trim35*, respectively. The third multigene set is composed of sequences that lack counterparts in tetrapods, the *fintrims* (*ftr*) [21]. These different gene expansion events explain the high *trim* numbers observed in fish compared to human.

Interestingly, the repertoire of class IV genes was also more divergent between zebrafish and pufferfish than for the other classes. Indeed, the multigenic class IV *trim* subsets contains much less genes in pufferfish compared to zebrafish: 12 *ftr* for 89, 6 *trim35* instead of 37 and 10 btr instead of 33. Additionally, several genes including *trim106-109* and *trim25* were absent from pufferfish while found in zebrafish.

**Figure 3:**
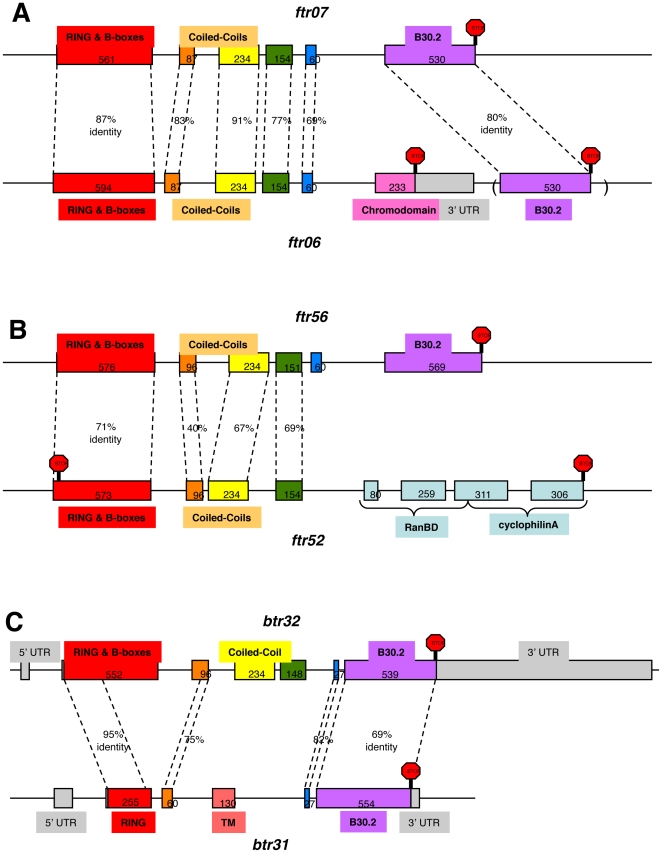
Three different ways to generate TRIM proteins with new domains. For these three cases observed in the zebrafish genome, the new gene is shown on the bottom of panel, and is compared with a closely related, typical member of the *ftr* or *btr* family illustrated on top. Percentages of identity refer to DNA sequences. Rectangles represent exons, numbers refer to nucleotides of coding sequence, stop included. Introns not to scale. A: the *ftr06* gene, contained within a large cluster of tandem *ftr* genes on chromosome 2, encodes a protein with a C-terminal chromodomain instead of a B30.2. This is due to the insertion of a single chromodomain-encoding exon just upstream of the usual exon 6. The previous B30.2 exon, shown in parenthesis, is still present downstream, nonmutated, but is not included in the chromodomain-encoding transcript. B: the *ftr52* gene, isolated on chromosome 9, encodes for a TRIM protein with a C-terminal RanBD/cyclophilin A domain instead of a B30.2. In this case, the new C-terminal domain is encoded by multiple exons; no B30.2-encoding sequence can be detected in this genomic area. C. The *btr31* gene, located on chromosome 19 tandemly to its close relative *btr32*, encodes for a protein with the typical N- and C- ends of bloodthirsty-like proteins, but the B-boxes and the coiled-coil regions in the middle have been replaced by a transmembrane domain.

Most remarkably, three instances of gain of domain were also detected (Figure 3). The *ftr06* gene acquired a C-terminal chromodomain via the insertion of a single exon between the original 5^th^ and 6^th^ exons (Figure 3A). Thus, although the sequence encoding for a B30.2 domain is still present in the genome downstream of the chromodomain, it is not included in the transcribed gene, as established previously by RACE analysis ([21] see sequences # AM941366 and AM941342). Along the same line, just downstream of the *ftr52* gene, one can find a Ran binding-domain (RanBD) and a cyclophilin A (CypA) domain, encoded by four exons, while no B30.2 domain can be detected in this genomic region (Figure 3B). Such a configuration could happen in one single step by the insertion of a piece of DNA containing exons 1 to 4 of a *ftr* gene within a pre-existing *RanBP2*-like gene. *Ftr52* was believed to be a pseudogene since it contains a predicted stop codon in the N-terminal RING-encoding region (found on the previous and current genomic assemblies). To test whether *ftr52* was effectively transcribed into a *trim* mRNA, we PCR-amplified cDNA from AB fish with primers upstream of the RING and downstream of the CypA regions, and cloned and sequenced the product (accession number JF295002). The retrieved sequence does correspond to a properly spliced transcript that would encode a RBCC-RanBD-CypA protein if not for a premature stop codon in the N-terminal exon. This stop codon is identical to the one found in the current genomic sequence, derived from a Tü strain fish. We PCR-amplified genomic DNA of several independent AB and Tü zebrafish (the most used “wild-type” laboratory strains) and found this stop codon in all products. A slightly more complicated sequence of events took place to generate the *btr31* gene that encodes a protein with a predicted TM domain between a N terminal RING domain and a C terminal B30.2 domain, while B-Boxes and the Coiled Coil have disappeared. This gene is clearly a product of recent duplication of a *btr* gene, and is most similar to its neighbour *btr32* that possesses the *bona fide* domains. The genesis of *btr31* would require at least two genomic rearrangement events: the replacement of exons 3 and 4 by a DNA stretch containing a TM-encoding exon, and the deletion of the end of exon 1; however the gene structure is confirmed by several ESTs (i.e., EH489524 and EH515884), excluding an assembly artefact. Similar to the first subset of trim genes, the loss of the specific C-terminal domains was also frequently observed in pairs of co-orthologues. Such events of gain of domain were not found in the pufferfish.

### *trim39/btrs* and *trim35*-related multigene families derive from ancient duplications

Fish genomes contain three large multigene subsets of class IV trims: *finTRIMs* (*ftr*s), bloodthirsty-like trims (*btr*s) and Hematopoietic lineage switch-5/trim35-like trims (*hltr*s). In an attempt to understand the selective constraints that give rise to such large families, we analyzed the diversity of the *ftr* family from a prior study [21] and extend this analysis to include *btr* and *trim35*/*hltrs* genes.

**Figure 4:**
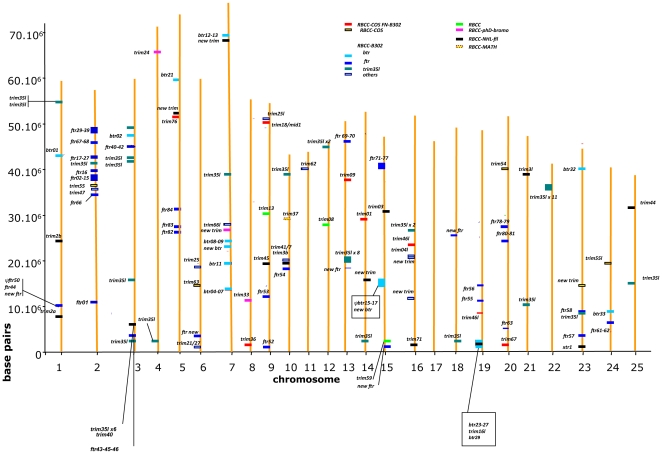
Genomic location of zebrafish *trim* genes. *trim* genes are depicted in different colors according to each *trim* class: class I in red, class II in yellow and boxed, class IV in blue, class V in green, class VI in pink, class VII in black, class VIII in yellow and red, class IX in orange. The different *trim* subsets belonging to class IV are indicated in shades of blue. This representation is based on the Zv8 assembly. RBCC: Ring-Bbox-Coiled Coil; COS: C-terminal subgroup one signature; FN3: Fibronectin, type III; B30.2: PRY/SPRY domain; PHD: Plant Homeo Domain; NHL: NCL-1, HT2A and Lin-41 repeat; Filamin: named from the protein Filamin; Bromo: acetylated lysine binding domain; ARF/SAR: from ARF and SAR GTP binding proteins; Pyrin: a member of the six-helix bundle death domain-fold superfamily; TM: transmembrane; Math: meprin and TRAF homology domain; Chromo: CHRromatin Organization Modifier domain.

**Figure 5:**
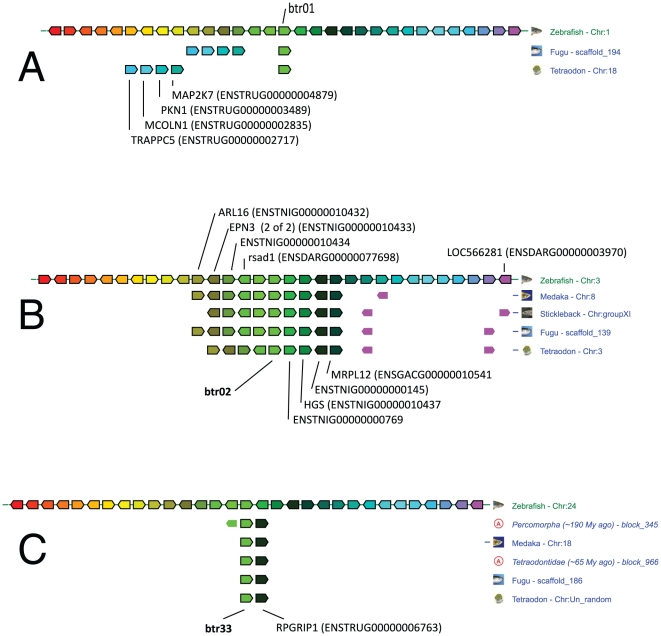
Group of conserved synteny around *btr* 01, 02 and 33. Synteny groups were determined from Ensembl assemblies using the genomicus database and browser (http://www.dyogen.ens.fr/genomicus-59.01/cgi-bin/search.pl) [85]. The figure is edited from the PhyloView taking *btr* 01 (A), 02 (B) and 33 (C) genes as references. The reference *btr* gene and its orthologues are shown in light green over a thin vertical line, and are indicated in bold.

The *btr* family, orthologous to *trim39*, has been previously reported in zebrafish and other teleosts [21]. These genes were named «bloodthirsty related genes» or *btr* from the first described member of the family, involved in erythropoiesis, *bloodthirsty* [35]. The *btr* genes are relatively dispersed in the zebrafish genome but do not colocalize with the *ftr* clusters. *btr* clusters are found on chromosomes 7, 15 and 19 (Figure 4). As previously seen for *ftr*, a minority of *btr* genes are part of synteny groups conserved in zebrafish, pufferfish and in other fish. *btr1* (chr1), *btr2* (chr3) and *btr33* (chr24) belong to gene sets found in conserved synteny (Figure 5). Additionally, *btr* genes located on zebrafish chr5 and 15 are linked to the paralogous markers encoding the alpha-crystallins *cryabb* and *cryaa* respectively, suggesting a common origin for the corresponding regions. Thus, at least the *btr* that are involved in conserved syntenies were produced by regional and global duplications which occurred relatively early during fish evolution. The other *btr* constituting clusters are probably more recent.

**Figure 6:**
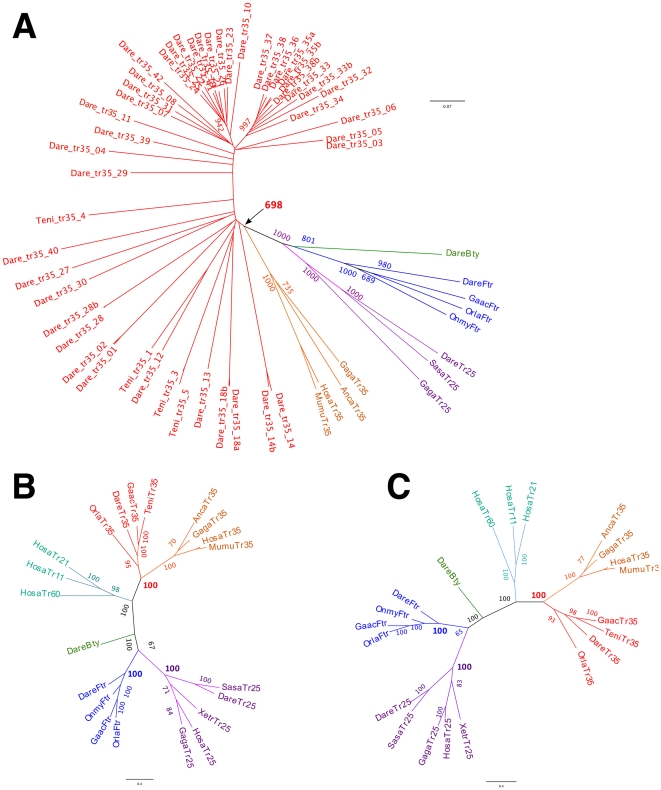
Fish counterparts of *trim35* constitute multigenic subsets. (A) Distance tree produced by ClustalW (Neighbor joining; boostrap = 1000) for the zebrafish TRIM35/HLTR sequences and representative TRIM sequences from other species. Relevant boostrap values are indicated. Separate phylogenetic analyses of the RBB (B) and B30.2 (C) regions of TRIM35 and other representative TRIM using Clustalw (Neighbor joining; boostrap = 1000). The same analyses were performed with PHYML and led to consistent trees. Sequences integrated into the trees: DareBty: zebrafish bloodthirsty (NP_001018311); DareFtr: zebrafish fintrim (XM_692536); GaacFtr: stickleback fintrim; OrlaFtr: medaka fintrim (ENSORLP00000003320); OnmyFtr: rainbow trout fintrim (AM887799); DareTr25: zebrafish trim25 (NP_956469); SasaTr25: salmon trim25 (gene index TC35355 accessible at http://compbio.dfci.harvard.edu/); GagaTr25: chicken trim25 (XP_415653); XetrTr25: Xenopus tropicalis Trim25 (Ensembl Xenopus genome scaffold255: 821309_819660); HosaTr25: human Trim25 (Q14258); GagaTr35 : chicken trim35 (ENSGALP00000026735); AncaTr35: lizard trim35 (ENSACAP00000002320); HosaTr35: human trim35 (NP_741983.2); MumuTr35: mouse trim35 (ENSMUSP00000022623); GaacTr35: stickleback TRIM35 (ENSGACP00000004694); OrlaTr35: medaka Trim35; TeniTr35: pufferfish Trim35; dareTr35: zebrafish Trim35-8 (ENSDARP00000064945); HosaTr21: human Trim 21 (NP_003132); HosaTr11: human Trim 11 (NP_660215); HosaTr60: human Trim 60 (AAI00986). The IDs of the other TRIM35 sequences from zebrafish used in (A) are available in Figure S1.

**Figure 7:**
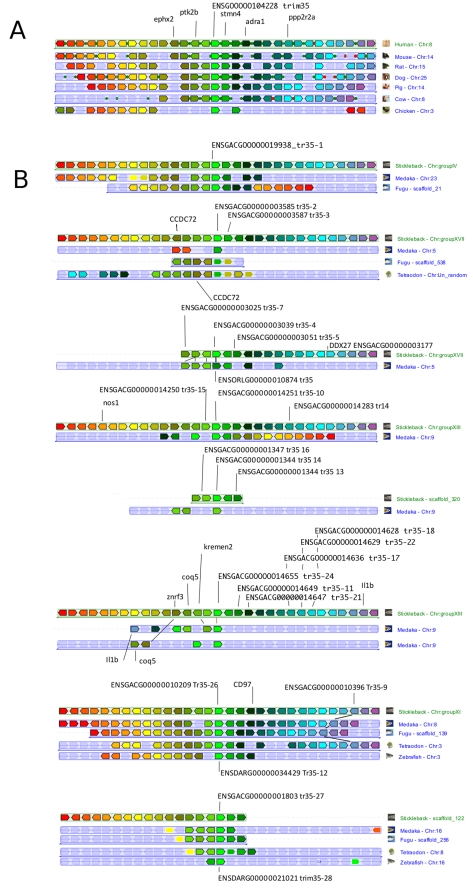
Group of conserved synteny around *trim35* genes and gene clusters. Synteny groups were determined from Ensembl assemblies using genomicus database and browser (http://www.dyogen.ens.fr/genomicus-59.01/cgi-bin/search.pl) [85]. The figure is edited from the PhyloView taking the human *trim35* (ENSG000000104228) as a reference (A), or taking the stickleback *trim35-01*, *trim35-02*, *trim35-04*, *trim35-10*, *trim35-14*, *trim35-24*, *trim35-26* and *trim35-27* genes as references (B). The reference gene and its orthologues is shown in light green over a thin vertical line and is indicated with its Ensembl ID.

Thirty-seven *trim35/hltr* genes were identified in the zebrafish genome (Zv9 assemby), all containing a B30.2 domain. In contrast, only six *trim35/hltr* were found in the pufferfish genome. When all zebrafish *trim35/hltr* sequences were included in a distance tree with representative genes from class IV, they grouped in a specific cluster, confirming that they constitute a distinct subset in the *trim* family (Figure 6A). Separate phylogenetic analyses were performed for RBB and B30.2 regions using NJ (Figure 6B &6C) and PHYML. These analyses indicate that the fish TRIM35/HLTR sequences group with the reptile and mammalian TRIM35, while its closest relatives TRIM21, 11 and 60 as well as FTR and TRIM25 determine distinct clusters each supported by high boostrap values in phylogenetic trees. Both RBB and B30.2 trees are congruent and strongly suggest that fish *trim35/hltr* genes are good co-orthologs of their mammalian unique (i.e. non-duplicated) counterpart. This hypothesis could not be further validated by examining conserved synteny because the markers defining a conserved 2 Mbp-neighbourhood of *trim35* in tetrapods (Figure 7A) are not found in the same synteny group in teleosts. In contrast, more than 25 of the *trim35/hltr* genes found in the teleost stickleback (*Gasterosteus aculeatus*) are part of synteny clusters conserved in medaka (*Oryzias latipes*) and even in pufferfish (Figure 7B), indicating that some duplications predated the split between these lineages. Only the regions involving *trim35-12* and *trim35-28* have counterparts in zebrafish. In zebrafish, the multiple copies of *trim35/hltr* are scattered on 15 different chromosomes (Figure 4) and they are often grouped in clusters as previously observed for *ftr* and *btr* ([21], see above). In striking similarity with the *ftr* genes, the *trim35/hltr* genes involved in conserved syntenies are not found among the large sets of recently duplicated sequences represented by the distal branches in the phylogenetic tree.

### Positive selection of B30.2 domains of *btr* and *trim35* families

The B30.2 domain consists of two subdomains, PRY and SPRY and forms a distorted β-sandwich of two layers of antiparallel β-sheets [36], [37], [38]. The ß-strands are connected by six variable loops (VL) that define hypervariable regions and form the ligand-binding surface in TRIM5α. The loops also contain the Ig-binding regions in TRIM21 [39]. We observed earlier in FTR*s* hypervariable regions similar to those of TRIM5α [21], and we showed that they evolved under positive selection. To determine whether zebrafish *trim35/hltr* and *btr* share the same evolutionary pattern and show diversified regions in their B30.2 domains, these genes were subjected to a similar analysis calculating the Shannon entropy site by site (see Supplemental Figure S4).

**Figure 8:**
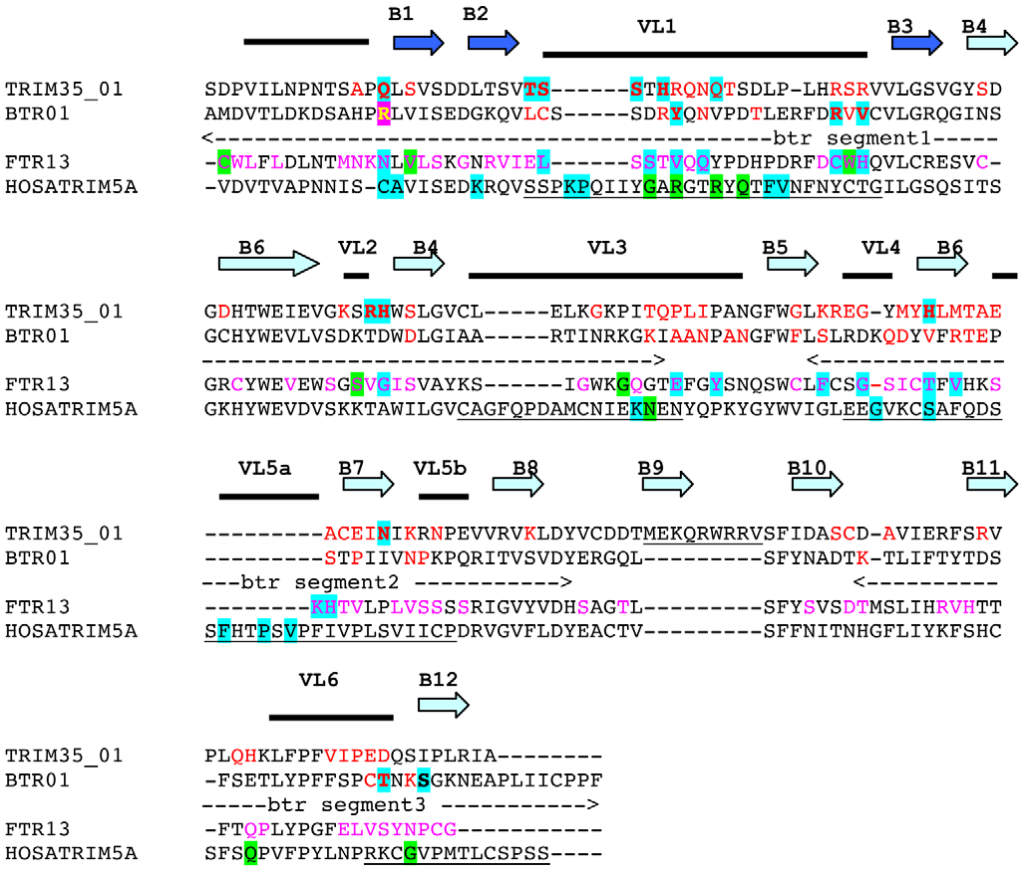
Positive selection in the B30.2 domain of BTR and TRIM35/HLTR. Distribution of hypervariable and positively selected residues in a multiple alignment of B30.2 domains from representative zebrafish BTR and TRIM35/HLTR, compared with a typical FTR sequence (Dareftr13: [GenBank: XM_695031]), and with TRIM5α (HosaTRIM5a). Hypervariable sites (shannon entropy >2) are indicated in red. Hypervariable sites previously described [21] are indicated in pink in the FTR13 sequence. The four hypervariable regions of the TRIM5α B30.2 domain are underlined. The variable loop-connecting strands of the domain are named VL1–VL6. ß-strands of the B30.2 domain are indicated by dark (PRY region) or light (SPRY region) blue arrows from [36]. Segments 1, 2 and 3 determined by the recombination GARD analysis in the BTR multiple alignment are shown under the BTR01 sequence. Positively selected sites (among zebrafish TRIM35/HLTR & BTRs: this study; among FTRs: [21] and among primate TRIM5α: [40] are boxed in blue when detected under models 2a and 8. Sites positive under M8 but not under M2 are boxed in green. In TRIM35/HLTR, Q (ß-strand 1) was detected under M2 not M8. In BTR, R (ß-strand 1) has been detected in the BTR analysis only under M8 with complete domain, not in segment 1. In BTR, S (ß-strand 12) has been detected only under M2a and M8 of BTR segment 3, not in the analysis using the complete domain. The detailed PAML results for each position under positive selection are available in Additional data file 8.

The distribution of variable sites in TRIM35/HLTR and BTR is remarkably consistent with the patterns observed for FTRs and for TRIM5α: 39 among 59 and 26 among 37 hypervariable sites of TRIM35/HLTR and BTR, respectively, are shared with FTR (Figure 8). Variable regions corresponding to the loops joining the B30.2 domain ß-strands were retrieved, including those involved in the binding of the virus by TRIM5α. Interestingly, conserved variable sites were concentrated in the β2–β3 loop (VL1), which is responsible of retroviral binding specificity of TRIM5α and was identified as an evolutionary hotspot in TRIM5α and TRIM22 [40].

To test whether *trim35/hltr* and *btr* B30.2 domains evolved under diversifying selection in zebrafish, we used a test based on the estimation of synonymous (*dS*, silent mutations) and non-synonymous (*dN*, amino acid altering) substitution rates of all codons among a set of sequences: the ratio ω = *dN*/*dS* is an indication for negative (purifying) selection of deleterious changes (ω<1), neutral evolution (ω = 1), or positive (diversifying) selection when changes offer a selective advantage (ω>1). This approach is often used on paralogues to detect the accumulation of non-synonymous changes that suggests a positive selection driving the evolution of new functions following gene duplications [41], [42]. This method requires that the paralogue sequences are not too divergent i.e. that good quality multiple alignments can be easily produced. We verified that this condition was met for each dataset subjected to PAML analysis (Supplemental Figure S5).

B30.2 domain sequences of zebrafish *trim35/hltr* and *btr* were analyzed under different evolutionary models M1a, M2a, M7 and M8 by PAML. Positive selection was detected for ∼10% of sites of *trim35/hltr* under both M2a and M8, while for *btr* 4.5% of sites were positively selected under M2a and 8.5% of sites under M8 (Table 1). These results were validated by significant likelihood ratio test (LRT) with *p*<0.001 for both models for *trim35/hltr* and *btr* (see Supplemental Figure S6).

**Table 1:** PAML results.

Region^1^	n^2^	c^3^	Parameters in ω distribution under M2a^4^		Parameters in ω distribution under M8^5^	
TRIM35	38	122	ω_>1_ = 5.68162	p_>1_ = 0.09952	ω_>1_ = 4.13995	p_1_ = 0.10057
complete B30.2			ω_1_ = 1,000	p_1_ = 0.43562		p_0_ = 0.89943
			ω_<1_ = 0.19870	p_<1_ = 0.46485	p = 1.13670	q = 1.81193
TRIM39	25	171	ω_>1_ = 3.33719	p_>1_ = 0.04501	ω_>1_ = 1.99982	p_1_ = 0.08529
complete B30.2			ω_1_ = 1,000	p_1_ = 0.31535		p_0_ = 0.91471
			ω_<1_ = 0.19082	p_<1_ = 0.63964	p = 0.88108	q = 1.86498
TRIM39 B30.2	25	84	ω_>1_ = 3.96823	p_>1_ = 0.08146	ω_>1_ = 2.74731	p_1_ = 0.08977
Fragment 1–252			ω_1_ = 1.000	p_1_ = 0.39488		p_0_ = 0.91023
			ω_<1_ = 0.14824	p_<1_ = 0.52366	p = 0.89035	q = 1.87745
TRIM39 B30.2	25	30	ω_>1_ = 1.000	p_>1_ = 0.13938	ω_>1_ = 1.00000	p_1_ = 0.09928
fragment 291–393			ω_1_ = 1.000	p_1_ = 0.32559		p_0_ = 0.90072
			ω_<1_ = 0.19144	p_<1_ = 0.5350	p = 1.18020	q = 2.35230
TRIM39 B30.2	25	39	ω_>1_ = 2.21718	p_>1_ = 0.08741	ω_>1_ = 1.90649	p_1_ = 0.12627
Fragment 430–557			ω_1_ = 1.000	p_1_ = 0.12322		p_0_ = 0.87373
			ω_<1_ = 0.19452	p_<1_ = 0.78936	p = 1.42717	q = 4.37468

1 for sequence fragments, the numbers correspond with the position of first and last nucleotides in the alignment with excluded gaps.

2 n, the number of sequences in the alignment and tree.

3 c, the number of codons.

4 parameters determined under M2a with ω the ratio of non-synonymous rates (dN) and synonymous rates (dS) and p the corresponding proportion of sites for each ω-class.

5 parameters determined under M8 with ω the ratio dN/dS, the corresponding proportion (p_1_ = 1−p_0_) of sites and p- and q-estimates in the β(*p*,*q*)-distribution.

To investigate whether the estimation of positive selection under PAML was not perturbed by recombination between similar *trim* sequences during the evolution of the zebrafish genome, we re-analyzed our dataset with the algorithm PARRIS, which uses a partitioning approach to test whether sequences have been subjected to positive selection even if recombination occurred. Positive selection was still indicated by the LRT with p<0.001 for both TRIM35/HLTR and BTR. This indicated that whether or not recombination did occur, the B30.2 domains of TRIM35/HLTR and BTR have most probably evolved under positive selection.

To search for recombination sites, we used the program GARD, which subdivides a sequence alignment in putative non-recombinant fragments, infers phylogenies for each fragment and assesses the quality of the fit for these phylogenies. This comparison therefore determines if the fragments are derived from two different ancestor sequences due to recombination. No evidence for recombination was detected for *trim35/hltr*. In contrast, seven breakpoints were identified between *btr* sequences at the positions 262, 269, 271, 288, 399, 405 and 427 of the *btr* B30.2 multiple alignment (see the multiple alignment in Supplemental Figure S5 and Δc-AIC values in Supplemental Figure S7). These breakpoints suggested the existence of three segments of *btr* B30.2 domain where no recombination has occurred (see the segments 1, 2 and 3 in Figure 8). We detected positive selection in fragment one and three, with p<0.001 in the LRT under M1a–M2a and M7–M8 models. No positive selection was detected in fragment two (Supplemental Figure S8).

Finally, the specific sites where non synonymous changes accumulated were identified by a Bayesian approach using the complete gene set for *trim35/hltr* and the fragmented gene set for *btr*. For *trim35/hltr*, we found 12 sites under M2a and 11 sites under the more restrictive model M8. The majority of the sites fall in the predicted variable loops corresponding to those reported for TRIM21 and TRIM5α. For the *btr*s, we found 7 sites under both M2a and M8.

Hence, a significant number of sites showing hints of positive selection in *trim35/hltr* and *btr* B30.2 domains were located in the β2–β3 loop, at positions matching well those previously noted for *ftr* and for the same region in TRIM5α (Figure 8).

### Do fish *trim* genes colocalize with the MHC paralogs?

The Major histocompatibility complex (MHC) is a genetic region that plays a key role in self/non-self recognition and T cell responsiveness. The presence of Class IV *trim* genes in the MHC is a conserved feature in mammals and birds [43], [44], [45]. Based upon this feature we addressed whether the co-localization predates the split (>450 My) between fish and tetrapods. This gene dense region has an ancient history as the mouse and human genome contain four well established MHC paralogous regions of the MHC, that are believed to be the result of two whole genome duplications in the early evolution of vertebrates [46]. In teleosts, a variable number of global genome duplications followed by genome contraction and rearrangement events have “broken” the MHC into multiple regions in the genome of fish such as the MHC class I and II regions are on different chromosomes [47]. To trace the existence of an early association of ClassIV *trim* genes with the “primordial” MHC, it was therefore relevant to examine the different MHC regions and all of their associated paralogues in fish genomes.

A loose linkage of *ftr*, *btr* and the MHC or its paralogues has been previously reported in zebrafish [20]. The MHC regions and their paralogues also contain RBCC-B30.2 genes in another fish species that possesses fewer Class IV *trim* genes than zebrafish: in stickleback, *notch1.1, notch1.2 and notch3* are associated to 7 genes belonging to the *trim* Class IV grouping. Since in humans, *notch4* is found within the MHC and other *notch* genes in paralogous loci [46], this was the first indication that the linkage might be older than tetrapods. We therefore performed a systematic survey of the distribution of *trim* genes and MHC markers in the zebrafish genome, looking for a co-localization pattern. We searched the homologues of a set of classical MHC markers and their paralogues described in other vertebrates [48], [49], [50], [51], [52], [53] (See Supplemental Figure S9). Since it was not always possible to attribute the zebrafish homologues to one given member of the tetrad of MHC paralogues in humans [54], we defined “MHC neighbourhoods” as regions extending 5 Mb (size of the MHC proper) upstream and downstream from each MHC or paralogue marker. The MHC neighbourhood represented 376 megabases in zebrafish containing 7884 genes compared to 1187 megabases and 16072 genes for the rest of the genome. We then compared the numbers of *trim* genes that were located in these MHC neighbourhoods versus that of the rest of the genome (see Supplemental Figure S9); counting each cluster of tandemly duplicated *trim* genes as a single occurrence to avoid skewing of the analysis. Interestingly, the Class IV *trim* genes were significantly enriched in the MHC neighbourhoods (Independence χ^2^-test, p value = 0.0035), while no bias could be detected for the other *trim* genes (Table 2).

**Table 2:** Class IV trims genes are concentrated in the MHC and MHC paralogues.

	MHC neighbourhood	Rest of the genome	Total	Chi square test
Length (megabase)	376^1^	1187	1563	
Number of genes (total)	7884	16263	24147	
Number of Class IV *trim* 2	31	31	62	p = 0.0035
Number of other *trim*	10	23	33	NS

1 The results are based on the genome assemblies available at http://www.ensembl.org (release 58). The detailed calculations and a map with MHC and MHC paralogues considered in the analysis are available in SupplMat 8.

2 To avoid skewing the analysis by the numerous *trim* recently duplicated, we counted each clusters of *trim* genes as only one event From the Zv8 assembly of the zebrafish genome.

## Discussion

TRIMs are widely distributed in metazoans, and these intracellular proteins are involved in the regulation of multiple pathways. In this report, a systematic survey of *trim* genes was performed in zebrafish and pufferfish to examine the characteristics of this family in two fish species with different genome dynamics. The zebrafish genome is large (about 1600 Mb/24000 genes) and contains an abundance of repeated DNA elements [55] as well as many highly expanded gene families. In contrast, the pufferfish genome is compact (about 350 Mb/15000 genes) and the multigenic families are smaller than found in zebrafish, at least those involved in the immune system. The pufferfish belongs to *Tetraodontidae* in the vast group of percomorphs, and it is phylogenetically distinct from zebrafish, with an estimated 300 My of divergence [22]. We therefore attempted a comparison of an extensive versus a minimal repertoire of *trim* genes in teleosts to better understand their evolutionary histories.

We retrieved a large number of *trim* genes in both species, representing almost all the classes defined by Short and Cox [3] in human. Our data indicate that the main TRIM classes were already defined in the common ancestor of fishes and tetrapods. However, a few genes show specific features that illustrate the evolutionary pathways leading to the generation of new *trim* classes. There is only one class III *trim* gene in humans (*trim42*) with orthologues in amniotes but not in fish. However, if class III genes are defined by domain organization alone (RBCC-FN3), fish do possess a class III *trim* gene, which is one of the two co-orthologues of *trim46*, a class I gene (RBCC-FN3-B30.2). One can then hypothesize that the human *trim42* itself derives from a class I *trim* gene through an ancient event involving the loss of the B30.2 domain. In the same line, the frequent loss of various C-terminal domains led to the birth of new class V *trims* found in zebrafish (e.g. *trim25like*, *trim54like*, several *ftr*s). This is also likely to be the case for some human class V *trim* genes; a relatively recent origin by such a mechanism would explain why few human members of this class have fish counterparts.

At the N terminus, loss of the RING domain is also observed in several instances (*trim24like*, *trim32*, *trim1* and *trim2like*). As described above, this event results in truncated *trim*-like genes, but could not result from the deletion of an entire exon, which suggests that the loss of the RING was positively selected. In fact, such events can be sometimes dated before the split between the pufferfish and zebrafish lineages, or some are even much older such as *trim16* that is retrieved in fish and tetrapods. The loss of B Boxes and Coiled Coil is also sometimes observed – mainly in Class IV genes - but does not seem to be fixed as easily.

In contrast, insertions of single- or multi-exon domains downstream of a RBCC module were found to generate new *trim* configuration in the zebrafish genome (i.e. *ftr06*, *ftr52*, *btr31*). When this occurred is unknown, but the insertion of the chromodomain in the *ftr06* gene appears to be recent, considering the dynamics of the *ftr* family and the absence of inactivating mutations in the B30.2 exon displaced by the “usurper” exon. Whether this change has a functional consequence for the encoded gene remains to be tested experimentally; a detailed phylogenetic reconstruction in close relatives of the zebrafish would therefore be informative. As chromodomains are involved in chromatin remodelling such a protein would be expected to regulate gene expression; similar functions have been described for TRIM proteins with a C-terminal bromodomain, structurally distinct from chromodomains but with comparable functions. In contrast, the replacement of the B30.2 exon of *ftr52* by exons encoding a cyclophilin A (CypA) domain could have given rise to a TRIM protein with affinity to different viral proteins, because most remarkably, TRIM5-cypA proteins have also appeared at least twice independently (by retrotransposition of a cypA sequence in the *trim5* locus) in the primate lineage, leading to proteins with demonstrated anti-retroviral activity involving capsid binding by CypA [56], [57], [58], [59], [60], [61]. In spite of this, no *trim* gene with a CypA domain has been reported in humans or in non primate species with a fully sequenced genome. The early stop codon found in the zebrafish *ftr52* gene leads us to speculate that although such a domain combination may provide a transient benefit against some viral infections, it may have some drawbacks that impairs its definitive fixation in a lineage. For *btr31*, the recombination events led to a unique configuration RING-TM-B30.2 where a membrane separates a RING and a B30.2 domain, which has completely unknown functional consequences.

Domain organization, sequence similarity and phylogenetic analyses indicate that one or two orthologues of multiple human *trim* genes that belong to classes I, II, V, VI, VII, VIII, IX i.e. to the “group 1” defined by Sardiello *et al.* [6] are present in both zebrafish and pufferfish. Often, when two co-orthologues are found for such genes, one of these has lost the C-terminal domain, while the other has retained the complete domain organisation and thus probably constitutes a true functional counterpart. This notion is also supported by similar expression patterns that were observed for genes selected in this category in vertebrates. The correspondence between the repertoires of “group 1” *trim* genes in zebrafish, pufferfish, and mammals indicates that strong purifying selection pressures were exerted to keep one (or few) copy(ies) of these genes in vertebrate genomes, illustrating their key functions in the basic biology of the cell. This is in sharp contrast with the evolutionary pathway of the Class IV RBCC-B30.2 *trim* genes.

The RBCC-B30.2 *trim* genes from Class IV represent unique sets in the different species of mammals and other tetrapods examined in detail by Sardiello and colleagues [6]. Our survey of zebrafish and pufferfish *trim* genes generally confirms and extends this conclusion. Most human ClassIV *trim* genes have no counterpart in the zebrafish or the pufferfish, and fish possess many ClassIV *trim* genes that do not exist in human nor in the mouse. Another feature of Class IV *trim* genes that was well exemplified in our previous report on *fintrim* [21] is their propensity to expand into multigenic subsets. In this study we demonstrated that fish possess in fact three multigenic subsets of *trim* genes all belonging to the Class IV: *ftr* (i. e. *fintrim*), *btr* (i.e. *bloodthirsty-related trim/trim39*) and *trim35/hltr*. The number of *ftr*, *btr* and *trim35/hltr* is different between fish species belonging to distant families, indicating different degrees of expansion. This is particularly striking from the comparison of zebrafish with expanded subsets and pufferfish with a “minimal” repertoire. This contrast likely reflects the high level of genomic rearrangement of the zebrafish genome – as indicated by short conserved synteny blocks compared to other fish versus mammals – and the strong compaction of the tetraodon/fugu genome [26], [62]. However, functional data would provide a better understanding of these sharp differences of class IV among teleosts. Besides, this underlines the strong constraints that maintained the conservation of *trim* belonging to the other trim classes (the “group 1” defined by Sardiello *et al.*) in different lineages. Some members of *ftr*, *btr* and *trim35/hltr* are part of conserved synteny groups conserved among teleosts, showing that their initial emergence and subsequent diversification is ancient in the evolution of teleosts and predates the differentiation of the main fish lineages. Consistently, these genes involved in syntenies appear to be the most ancient genes in their subset. They branch close to the basis of their multigenic subset in phylogenetic trees and generally do not belong to large genomic clusters. This is the case for *ftr 82/83*, *btr-1 & -33*, *trim35-12 & 28*. In contrast, the genes composing large genomic clusters such as zebrafish *ftr* on chromosome 2, are not included in conserved synteny groups and probably represent more recent, lineage-specific diversification events. The mechanisms for the amplification of *trim* are likely different for *ftr*, *btr* and *trim35/hltr* within a species: for example in medaka the *trim35/hltr* expansion occurred by duplication, while *ftr* expansion involved retrotransposition. Interestingly, only three sets of class IV genes are retrieved as multigenic groups in any fish species for which a complete genome assembly is available. The diversification of *ftr*, *btr* and *trim35/hltr* therefore appears to be rooted in ancient duplication events, followed by parallel diversification processes, which reflects similar functional constraints in different fish lineages. Multiplication of some class IV *trim* genes has also occurred in mammals, albeit to a smaller scale; thus, human *trim5*, *trim6*, *trim22* and *trim34* likely result from such a duplication event, while in cow, the *trim5* gene has further expanded into eight tandem copies, five of which encode a functional protein [40].

To date, functions of the multiple Class IV fish *trims* are still largely unknown and do not provide an obvious explanation for their extensive expansion. A non-redundant role in erythropoiesis has been reported for *Bloodthirsty (bty)* [35] which is quite difficult to understand in the context of the large multigene *btr* family - it is also noteworthy that this role is deduced from morpholino-based transient inactivation in embryos and the original *bty* gene is not found in the current zebrafish assembly zv9 (www.ensembl.org; Tübingen background). The closest zv9 gene is *btr18*, and it remains to be established if *bty* is unique to the original genetic background used by Yergeau *et al.* or is an allelic variant of a zv9 gene. At least some finTRIMs are induced by IFN and virus infection in rainbow trout [21], [63], and a *btr* is upregulated by poly I∶C in Atlantic cod [64]. In fact, these *trim* genes were not only duplicated many times, but also diversified after gene expansion with an accumulation of non synonymous changes. Thus, apparent signatures of diversifying selection were found in the β2–β3 loop in the B30.2 domain of *btr*, *trim35/hltr* (this study, Table 1) as previously reported for *ftr* [21] in zebrafish. Interestingly, the B30.2 domain – especially the β2–β3 loop - was subjected to a strong diversification in primates and accounts for the species-dependent retrovirus restriction of TRIM5α in the different species [18], [19], [65]. Moreover, several copies of *trim5* can be found in the genome of certain species such as cow [40]. Considering the importance of *trim* genes for antiviral immunity [66], [67] and the role of the B30.2 domain, we believe that virus sensing/restriction may be the driving force in the diversification of the fish *trim* multigene subsets under positive selection. However, the approach we followed to find sites under positive selection may lead to false positive, and accumulation of non synonymous changes does not necessarily imply functional changes [68]. Experimental evidence – for example of multiple B30.2/virus binding - would be required for a definitive proof of the functional impact of B30.2 diversification.

Our simple analysis of localization of *trim* genes relatively to genes of the MHC and MHC paralogues would have to be complemented by a detailed phylogenomic analysis of these regions through the whole vertebrate evolution from lamprey and sharks to fish and mammals. This will become possible with the publication of good quality genomes. However, the co-location pattern that we report suggests that *trim* and B30.2 are associated with the MHC and MHC paralogues in fish as well as already reported in mammals and birds [43], [44], [45]. Could it be for the benefit of immunity? An interesting question then would be to determine if the B30.2 domain was first associated to the ancestral MHC as a part of a pre-existing Class IV TRIM molecule. The existence of *trim*-like genes with canonical B30.2 domains in *Branchiostoma* (Cephalochordates), *Drosophila* (Arthropods), *C. elegans* (Nematods) *Nematostella* (Cnidarians) and *Trichoplax* (Placozoa) ([20] and unpublished observations) indicates that genes resembling Class IV *trims* are probably very ancient and could have been inherited from a common ancestor to vertebrates and these different groups of invertebrates. Considering the role of the B30.2 domains in mammals, we propose that ancestral Class IV TRIMs participated in defence and were part of a gene complex, the proto MHC, equipped in genes selected for processing (and later presenting) viral peptides. Indeed, intense duplication is typical of genes families involved in immunity. Such genes belonging to different families have diverged rapidly and independently within different classes of organisms in function of the pressures exerted by the pathogenic environment [69], [70], [71], [72], [73], [74]. Besides, *trim* genes may have been kept in a genomic cluster with proteasome components because they were involved in targeting virus particles to the ubiquitin-dependent proteasome system in a manner analogous to LMP/TAP genes that form a tight cosegregating unit in practically all vertebrates. This simple antiviral axis might have been very ancient and could have participated in the establishment of a proto-MHC selected for proteasome-mediated destruction of virus proteins and therefore production of peptides to which the antigen presenting machinery would be added later in evolution.

In support of this hypothesis one can remark that several class IV TRIM such as *trim11* and *trim17*, are located in human MHC paralogous regions and participate in the ubiquitin-proteasome system [75], [76]. The binding of TRIM5α to the retroviral capsid induces a rapid degradation of TRIM5α by the proteasome, providing an additional link between TRIM-dependent virus restriction and proteasome activity [77]. Another class IV TRIM involved in the ubiquitin-proteasome axis is TRIM21 that binds Ig constant region with very high affinity and targets viral particles coated with antibodies to the proteasome [78].

Given the abundance of viruses in the aquatic environment where early metazoa developed, the necessity for diverse protective measures against viruses certainly played a major role in shaping the immune system. The recruitment and diversification of IgSF TCR-like antigen receptors from proteins used by viruses to enter cells would be a good example of the consequences of such measures [79]. Similarly, the *trim* connection with the MHC could be a remnant of the early steps towards the construction of an adaptive immune system with associative recognition (TCR, MHC-peptide) by recruitment of antiviral primary defence systems. In the genomes of modern species, the *trim* family provides a good model to study the evolution of multigene families and functional diversification. The identification of the ligands and functions of such diversified subsets should provide new insights on the molecular pathways developed in the main vertebrate lineages.

Our survey of fish *trim* genes in two fish species identifies subsets with very different evolutionary dynamics. Thus, *trims* encoding RBCC-B30.2 proteins show the same evolutionary trends in fish and tetrapods: they are fast evolving, often under apparent positive selection, and they duplicate to create multigenic families that can be very large such as zebrafish *ftr*s. Among these multigenic subsets, we could identify several new combinations of domains, which epitomize how new *trim* classes appear by domain insertion or exon shuffling. Finally, *trim* encoding RBCC-B30.2 proteins are preferentially located in the MHC and in MHC paralogues, which suggests that such *trim* genes with a B30.2 exon may have been part of the ancestral MHC.

## Materials and Methods

### Identification of a complete array of genes from the *trim* family in zebrafish

Zebrafish *trim* genes – defined as encoding proteins with a RING-B-Box-Coiled Coil (RBCC) motif – were searched in the Zv8 genome assembly available at http://www.ensembl.org/. The survey was later updated from the current assembly (Zv9, made available at the end of 2010). The new assembly Zv9 did no show any major change in number, structure or location of *trim* genes. Both lists are given in Supplemental Figure S1 for an easier comparison with previous reports.

Several strategies were followed in parallel to try to get a complete list of zebrafish *trim*. First, all zebrafish ensembl proteins with a motif RING (ipr IPR001841) or B box (ipr IPR000315) were extracted using the biomart tool, and the intersection of the two lists kept as a first set of trim sequences (Set#1). The ensembl Ids, annotation, locations and status were also extracted. Second, the protein sequences belonging to the TRIM Ensembl families detected in zebrafish (ENSFM00300000079125, ENSFM00400000131833, ENSFM00250000004079, ENSFM00250000005797, ENSFM00390000126422, ENSFM00500000272256, ENSFM00500000271543, ENSFM00500000272036, ENSFM00390000126385, ENSFM00250000006428, ENSFM00250000001082, ENSFM00500000270185, ENSFM00250000001642, ENSFM00400000131788, ENSFM00250000004429, ENSFM00250000008223, ENSFM00500000287404): were collected and combined with the set #1 (set#2). Third, the zebrafish ensembl orthologs of all human trim gene were collected; the human orthologue of each gene was then checked, and this information was used to annotate the genes previously identified.

To compare the sequences to our previous work on two multigenic *trim* subsets performed on the zebrafish Zv7 assembly – the *fintrims* and the bloodthirsty-related (*btr*) *trim*s – we used the TBLASTN program at http://www.ensembl.org/ to align the FTR and BTR protein sequences with the current genome assembly. We also compared the sequences of *ftr* and *btr* genes extracted from Zv7 to the current assembly. Using both alignment scores and hit location, the *ftr* and *btr* sequences were identified in the set#2. For the new *ftr* and *btr* present in Zv8 as well as for the *Trim35* multigenic family, sequences were manually edited from gene models available in both Ensembl and Genbank. When the *ftr* or *btr* genes were fully retrieved in Zv8, we kept our previous manual annotation rather than the Ensembl automatic assignment. For the other *trim*, the protein models from Zv8 and Zv9 were considered, and the most recent annotation available. Finally, the protein sequences corresponding to this *trim* list was subjected to a domain analysis using Interproscan. The sequences unassigned yet were then manually annotated one by one. Starting from each zebrafish *trim*, we searched for the possible orthologues and paralogues in pufferfish. The orthologues of each human and zebrafish *trim* were searched in the Ensembl database. All proteins including a RING and a B30.2 domains were also extracted, which confirmed that the previous list was comprehensive.

### Cloning of *ftr52* sequences

Transcript sequences were amplified from cDNA of pooled 5dpf AB larvae with AccuStar DNA polymerase (Eurogentec) using primers ATGAATTCGTGTAAATACAGCGAAATGGCA and ATGCGGCCGCACCTAGGCTCACAGCTG. A band of ∼2 kb was gel-purified, digested with EcoRI and NotI, and cloned in the pBK-CMV plasmid. The genomic region encompassing the RING-encoding domain was PCR-amplified with primers TACAGTGGCTCGTCAAGTGA and TGCACTCTTCATCCGTGTGA.

### Detection of positive selection in B30.2 domain

The dataset for positive selection analysis was prepared from *btr* and *trim35/hltr* sequences that were found on the Ensembl zebrafish assembly. Domains were identified by the web-based tool Simple Modular Architecture Research Tool (SMART) at http://smart.embl-heidelberg.de/. A multiple sequence alignment was made for each domain with ClustalW within the *MEGA*4 software and gaps were removed from the alignment.

The Codeml program of the Phylogeny Analysis by Maximum Likelihood (PAML) package [80], retrieved from http://abacus.gene.ucl.ac.uk/software/paml.html , was used for the detection of positive selection. The models M0, M1a, M2a, M7 and M8 were employed. The ratio of synonymous (*dS*) to non-synonymous (*dN*) substitution rates, *ω = dS/dN*, is determined by the program. We used the site-specific model that allows ω to vary among sites. The null models M0, M1a and M7 do not allow the existence of positively selected sites (ω>1), while the alternate models M2a and M8 allow ω>1. M8 follows a beta(*p*,*q*)-distribution and is less stringent than M2a. Within the models, a Maximum Likelihood algorithm is used, whereby the sites are allocated under classes of different ω probabilities. Sites allocated under the class with ω>1 are considered as being under positive selection and were identified by a Bayes Empirical Bayes (BEB) analysis. Significance of outcome was confirmed by a likelihood ratio test (LRT). In the LRT we took twice the difference in log likelihood (2ΔlnL) between the nested models and used the chi-square test with the degrees of freedom (df) being the difference in free parameters between the two models (M1a vs. M2a and M7 vs. M8). Tests were considered positive when *p*<0.001. Sites identified by BEB with a posterior probability higher than 95 percent were considered significant.

### Analysis for recombination

To test for interference of recombination on the PAML results, we implemented a test by the algorithm PARRIS [81]. Under PARRIS, the PAML models M1a–M2a are employed with incorporation of site-to-site variation in synonymous substitutions rates and partitioning of data. We used the codon model for evolution GY94×HKY85 and a discrete distribution of three bins for synonymous and for non-synonymous rates. Significance of results was tested by a LRT.

We detected recombination breakpoints by the algorithm GARD [82]. We used the HKY85 model with general discrete distribution of rates across sites. We performed two screenings, for 2 or 20 breakpoints. The detection was validated by corrected Akaike's information criterium (c-AIC) for best-fitted model selection. Both PARRIS and GARD are integrated in the HyPhy software package that was retrieved from http://www.hyphy.org.

### Fish, RNA isolation and real time quantitative PCR

RNA was extracted from either single fish or pooled organs from five to ten two-year old zebrafish of AB background. All the animal experiments described in the present study were conducted at the Institut Pasteur according to the European Union guidelines for the handling of laboratory animals (http://ec.europa.eu/environment/chemicals/lab_animals/home_en.htm) and were approved by the Institut Pasteur animal care and use committee and by Direction Sanitaire et Vétérinaire de Paris under permit #A-75-12-22. Dissected organs, or entire fish cut in 3 mm pieces, were stored for a few days in RNALater (Ambion) before RNA extraction using TriZol (Invitrogen). DNA contaminations were removed by DNAse I treatment followed by phenol-chloroform extraction; integrity of the resulting RNA was checked on an 2100 bioanalysis station with a RNA nano chip (Agilent). cDNA was generated using M-MLV H- reverse-transcriptase (Promega) with a dT_17_ primer. Quantitative PCR was then performed on an ABI7300 thermocycler (Applied Biosystems) using SYBR green reaction power mix (Applied Biosystems). The following pairs of primers were used:

*EF1α*: GCTGATCGTTGGAGTCAACA and ACAGACTTGACCTCAGTGGT

*trim1*: CAAAACCAACAGTCAGCCTTT and AAGAGCGTACCATGTAGAGG

*trim13*: CAGGTAGACAAACTTTGCGC and CAGTCCGACGGAAGAAAGTT

*trim25*: GAGCGGCGCTTCAAACAAAA and ATCAATTGCCAGCATGGCCT

*trim33*: GTTCCTACCTCGGTTCCTAA and GAATCGGCCTGGACATTACT

*trim54*: GGAGCATCAAGGACAATGGT and CTTCGTGCTCTGCAGGAATA

*trim59*: CTGGTGCAGAAAGATCGAGA and CTCGTAGGCCTGATTGAGAA

Quantifications were performed on triplicate wells, and taking into account the previously measured yield of the reaction as described in [83]. To normalize cDNA amounts, we have used the housekeeping gene *EF1α*, chosen for its high and stable level of expression over development and among tissues [84]. After calculations of *trim/Ef1α* transcript expression ratios, data have been normalized to the average expression in entire fish (using the geometric mean of the results obtained on the whole male and the whole female), to highlight which organs express higher or lower levels of a given gene compared to the rest of the body. Results are reported as mean ± standard deviation of the measured ratios.

## Supporting Information

Figure S1 A table listing the *trim* genes of zebrafish, with domain structure, annotations and genomic locations in Zv8 and Zv9 assemblies.
(XLS)

Figure S2 A table listing the *trim* genes of pufferfish, with annotations and genomic locations.
(XLS)

Figure S3 A table showing the correspondence between *trim* genes in zebrafish and pufferfish, allowing an easy comparison of their domain structures.
(XLS)

Figure S4 A phylogenetic tree showing the evolutionary branching of the genes selected for the qRTPCR expression analysis.
(TIF)

Figure S5 Figure S5 shows the profiles of Shannon entropy calculated site by site from zebrafish *trim35/hltr* and *btr* alignments to determine the hypervariable regions, and the multiple sequence alignments used for positive selection calculations.
(DOC)

Figure S6 A table showing the results if of the likelihood ratio test (LRT) of positive selection of zebrafish *trim35*/*hltr* and *btr* B30.2 domains.
(DOC)

Figure S7 A table showing the results of the GARD program for recombination between zebrafish *trim35* and *btr* B30.2 domains.
(DOC)

Figure S8 A table that summarizes the positive selection analysis for the different regions of zebrafish BTR B30.2 domains.
(XLS)

Figure S9 A table listing *trim* genes and the zebrafish homologues of a set of classical MHC markers and their paralogues, with their genomic location.
(XLS)
