# Supplementary figures and images for: Origin and Evolution of TRIM Proteins: New Insights from the Complete TRIM Repertoire of Zebrafish and Pufferfish
Source: PLoS One. 2011 Jul 15;6(7):e22022. doi: 10.1371/journal.pone.0022022 (PMC3137616; doi:10.1371/journal.pone.0022022)

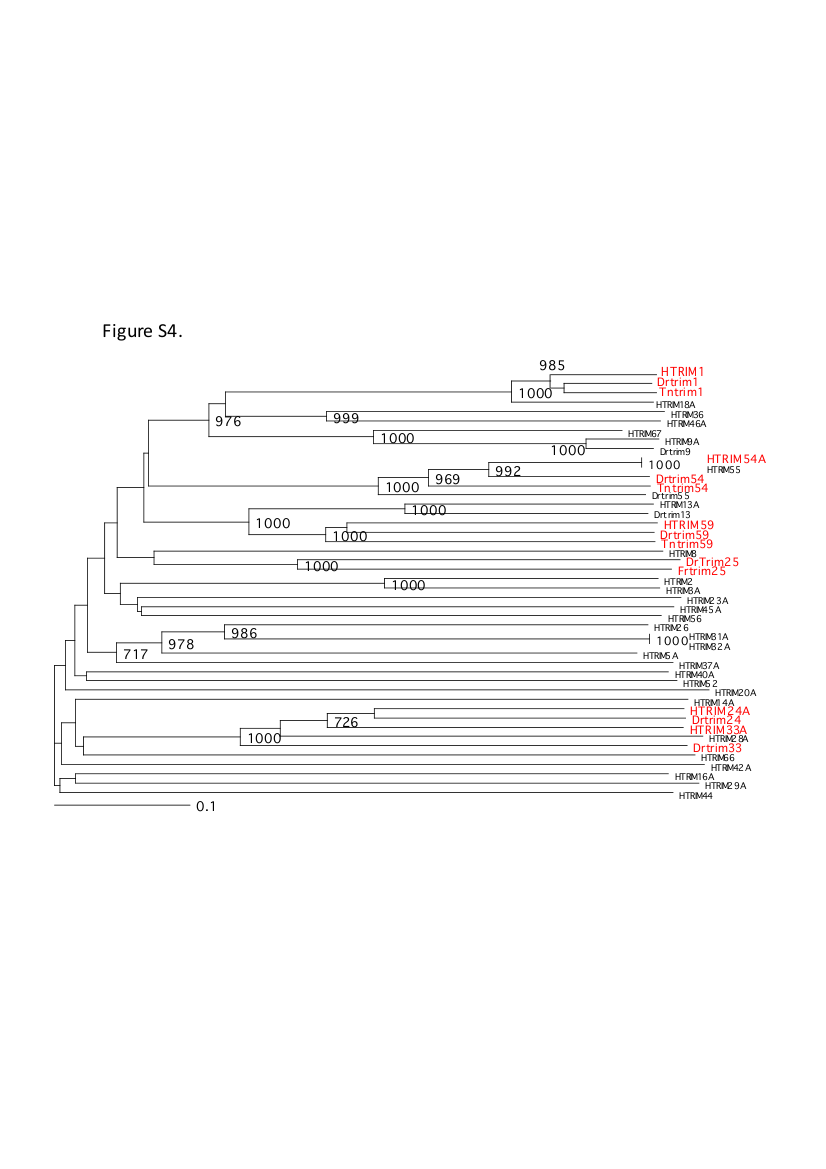

Supplement: Figure S4 — A phylogenetic tree showing the evolutionary branching of the genes selected for the qRTPCR expression analysis. (TIF) [file pone.0022022.s004.tif]
