# Supplementary material for: Origin and Evolution of TRIM Proteins: New Insights from the Complete TRIM Repertoire of Zebrafish and Pufferfish
Source: PLoS One. 2011 Jul 15;6(7):e22022. doi: 10.1371/journal.pone.0022022 (PMC3137616; doi:10.1371/journal.pone.0022022)
Supplement: Figure S5 — shows the profiles of Shannon entropy calculated site by site from zebrafish trim35/hltr and btr alignments to determine the hypervariable regions, and the multiple sequence alignments used for positive selection calculations. (DOC) [file pone.0022022.s005.doc]

FigureS5.

Shannon entropy of BTR B 302 domain alignment (pvs server)


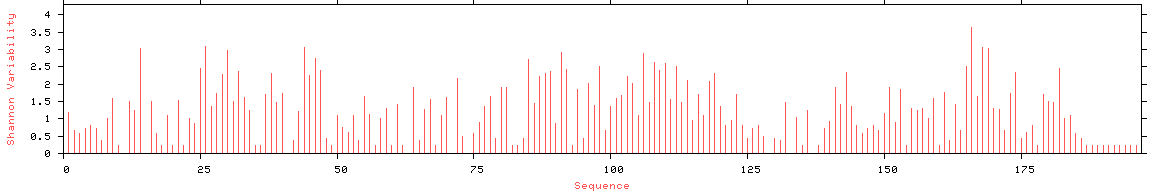


Shannon entropy of TRIM35 B 302 domain alignment (pvs server)


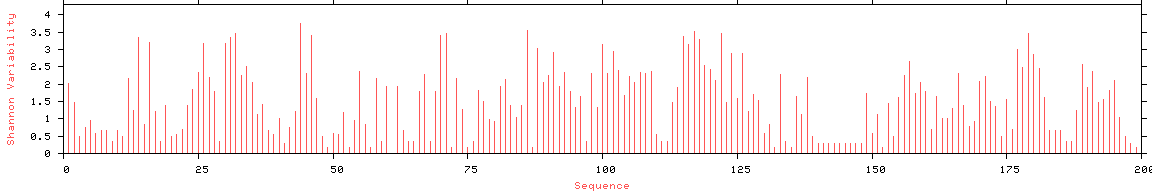


BTR

CLUSTAL W (1.83) multiple sequence alignment used for computing the Shannon entropy profile of btr B302 domains.

B302_BTR01 AMDVTLDKDSAHPRLVISEDGKQVLCSDRYQNVP--DTLERFDRVVCVLGRQGINSGCHY

B302_BTR11 AVDVTLDANTAHPRLILSENKKMVWCSENQQHVT--NHRERFDRVVCVLGREGFNTGRHY

B302_BTR02 SVDVNLNPRTAHAYLYISEDRKEVRHANKQQEVP--ENPKRFDRVINVMSKEAFRYGRHL

B302_BTR24 AVDVTLDPDTAHPDLILSDDGKRVRDGDIRQKLP--DKPQRFDYCVCVLGKEGFSSGRFY

B302_BTY AVDVTLDPDTAHPKLILSDDGKQVRCGDIRQELP--DTPQRFDYCPCVLGKEGFSSGRFY

B302_BTR23 AVDVTLDPDTAHVGLILSKDEKQVRLGEIIQKLP--DKSQRFEKCICVLGKEGFSSGRFY

B302_BTR25 AVDVTLDPDTAHPYLILSDDGKQVRDGDIEQKLP--DKPQRFDRCPCVLGKEGFSSGGFY

B302_BTR18 AVDVTLDPDTANPYLILSDDGKQVKHGDIKQKLP--DTPQRFDRYEDVLGKEGFSSGRFY

B302_BTR22 AVDVTLDPDTANPYLILSDDGKQVRCGDIEQELP--DKPQRFDKYEDVLGKEGFSSGRFY

B302_BTR20 AVDVTLDPDTAHPKLILSDDGKQVKHGDIRQKLP--NKPQRFDRYAMVLGKEGFSSGRFY

B302_BTR19 AVDVTLDPDTANPHLILSDDGKQVKHGDIRQNLP--DTPQRFDTCPCVLGKEEFSSGRFY

B302_BTR29 AVDVTLDPHTAHPELILSDDGKQVSCGDIWQKLP--NKLERFNRYLSVLGREGFSSGRFY

B302_BTR21 AVDVTLDPDTAHPELILSDDGKQVRDGDIIQKLP--NNPQRFDYSVSVLGKEGFSSGRFY

B302_BTR31 AVDVTLDHDTANPFLILSDDEKQVSLGHIERNVP--ENPERFNHTVSVLGKQGFSSGKFY

B302_BTR32 AVDVTLDPDTANPFLILSDDEKQVSHGDIEYDVP--EIPERFDYTVSVLGKQGFNSGKFY

B302_BTR15 AVDITMDPETAHPNLILSDDGKQVTNGDIKLELP--DNPERFSTCCCVLAKEGFNSGRFY

B302_BTR17 AVDVTLDPNTAHPKLILSDDGKQVTFGDKELKLL--NNPERFDCCPCVLAKERFSSGRFY

B302_BTR16 TVDVTLDPDTAYSKLILSDDRKQVTYGATVQKLP--ANPKRFDSCCSVLAKEGFNSGKFY

B302_BTR06 AVDVTLDPDTANPFLILTDDGKQVTHGDTNQNLP--NNPQRYDQCNTVLGKEGFSSKRFY

B302_BTR07 TVDVTLDPDTAHQKLILSDDGKQVSTEDTKQKVP--NNLERFDKCICVLGKEGFLTGRLY

B302_BTR05 AVDVTLDRDTAYPELILSDDGKEMIQGDVYQDLP--DIPERFDHCPSVLGREGFSSGRFY

B302_BTR04PS AVELTMDPDTAHPELLLSDDRKQVRVQDVEHELP--DIPERFDYCPDVLAKEGFSAGRFY

B302_BTR08 TVNVTMDPETAHPKLFLSEDDKQAEFGETRQPVP--DNPWRFNKCPSVLGKEGFCSGKFY

B302_BTR09 SVDVILDPDTAHPKLILSEDGKQVRYGNIKHDRP--GSDKRFENYIVVLGKEGFSSGRFY

B302_BTR33 AVDLTLDHDTANSWLAVSEDRKSVSDGNVERNFQ--NNTQRFDTAPCILSKEPISRGRSY

B302_BTR12 ASELILDPATAQRDLCLSEDGKQVRYEEQRKNSSNSDTPRRFSPALFVLAREGFSSGRHY

B302_BTR13 SVDVTFDPDTANPWLQLSEDGHQIRHLGSWQDLR--DAPERFDTVVIALGRQGFCSGRRY

B302_BTR30 LEDVSLNPVTAYPFLILSEDRKQLKRGEKLQFYR--NNTQRYDVWSCVLAKESFQTGRHY

:: :: :* * ::.: : *:. :.:: :

B302_BTR01 WEVLVSDKTDWDLGIAARTINRKGKIAANPANGFWFLSLRDKQDYVFRTEPSTPIIVNPK

B302_BTR11 WEVKVNGKTDWDLGVASHSCNRKGKIKVSPSHGYWFLSLRDKNNYAFRTEPPTVLHLSNK

B302_BTR02 WEVDVGDKTDWDLGVAKQSVNRKGKFTICPSNGFWTLSLKNGSQYVANTYPPTSFNLSHK

B302_BTR24 FEVQVKGKTDWDLGVVRESINRKGQITASASKGFWTVVLRNGIEYKACASPPVSLPVKVK

B302_BTY YEVQVKGKTDWDLGVARESINRKGKITLTPGNGFCTVWLRKENE---CFS----LSLKVK

B302_BTR23 FEVQVKGKTKWDLGVARESVDRKGEIRLCPSNGFWTVWLR-KNEYKALASPSVPLSVKVK

B302_BTR25 FEVQVKGKTDWTLGVVRESINRKGEITLTPSNGFWTVWLR-KNEYKALASPSVPLSLKVK

B302_BTR18 FDVQVKGKTDWSLGVVKESVDRKGEFALSPGNRYWTVWLRNGNGYEACADSPVSLSLRVK

B302_BTR22 YEVQVKKKTDWTLGVVRESVDRKGEITLSPGNGYWTVWLRNGNEYKFCADSPVSLSLKVK

B302_BTR20 FEVQVKGKTKWDLGVVRESVDRKGKITLRPSDGFWTVCLRNGNQYKAGADSPVSLSLKVK

B302_BTR19 FEVEMKGKNKWDLGVA---------------TGFWAVVLRNGNEYKACADSPVSLSVRVN

B302_BTR29 FEVLVKGKTDWTLGVARESVDRKGDIRVSPETGSWTVALINGNELSARADPPVLLSLRVS

B302_BTR21 YEVQVKGKIEWELGVARESVGRKGTYTLNPSNGHWTVVLRNGNEYRACTGPSVSLSLKVK

B302_BTR31 YEVQVKGKTDWTVGLARESINRKGQITPSPEKGFWTVWLRNGNQYEALDSPAVSLSLSTS

B302_BTR32 YEVQVKGKKEWDLGVARESISRKETNQLTPANGFWTMALINENEYLICDDPVVSFPQRAK

B302_BTR15 FEVQVKQKTDWDLGVVRGSANRKGKITEAPEDGYWAVAFRKGNQYQVFKSPTVSLSLRVK

B302_BTR17 FEVQVKEKTDWDLGVVRESINRKGDITAAPEAGYWIIMLRNENQYLAIDSSSVSLSLRVK

B302_BTR16 FEVQVKGKTDWDLGVARESVNRKGTITGSPVNGYWIIVLRNGSQYKARESPTVSLSLKVK

B302_BTR06 FEVQVKEKTKWDLGVASESVKRKGKITLSPQHGYWAVGLRNSDVYWAFDAPAVRLSVREK

B302_BTR07 FEVQMKGKTEWTLGVARESINRKGKITVGLQDGYWAVGLRNENEYWAYAAPAVRLSVREK

B302_BTR05 FEVKVKGKTEWILGVVRESINRKGKIKLSPQDGHWCVALMKGDQYWACADPAVSLNVK--

B302_BTR04PS FEVQVKGKTDWVVGVARESINRKGEITVNPQNGFWAVGLRNESEYKACTGPAVSLSLRVK

B302_BTR08 FEVQVKGKTEWDLGVARESVNRKGIITLSPRNGLWTLWLRNGAEYKACDCLSVSLCLKVK

B302_BTR09 FEVNVSGKTEWLLGVARESLNRKGEFFLSPNDGNWSLWLKDENKCEACESLTLSLSLKVK

B302_BTR33 WEVGVSGKTAWDLGVARKSVNRKGLVTLSPEDGYWAVCLRNGCEYRACNRESELLSLKSL

B302_BTR12 WEVDVGHKTAWTVGLARSSARRKGEIRLNPEGGFWCLWLKNG-EVKALTGSRVALHLTSL

B302_BTR13 WEVQVGEKDDWYLGVARGSVNRKGRISVSTTQGYWALAMKKGQEYRVSSSPPLLVSVEHK

B302_BTR30 WEVSVGENRDWKLGVVCESAQRKGLFDMTPAAGYYALWWSGNHLRALTAPSLSKVKVAGH

::* : : * :*:. : .

B302_BTR01 PQRITVSVDYERGQLSFYNADTKTLIFTYT-DSFSETLYPFFSPCTNKSGKNEAPLIICP

B302_BTR11 PQKMGLFVDYEKGQVSFYDVDAKMHIHTFM-DNFSETIYPFFSPCTNKNSKNEAPLVITP

B302_BTR02 PKRVSIYLDYDEGRVSFYCSDTGTHIYSFR-DSFTDKLHPILSPGRPHGEKNTAPLIISS

B302_BTR24 PQRVGVFVDYEEGLVLFYDVESSSHIYSFTSQTFTDKLHPLFSPCPNCAGKNSNPLIITP

B302_BTY PQRVGVFVDYEEGLVSFYDVESSSHIYSFTDQTFTDKLYPYFSPCPNYEGKNSNPLIITP

B302_BTR23 LQRVGVFVDYEEGLVSFYDVESSSHIYSFTGQTFTDKLYPFFNPCSNIDGNNSDPLIITP

B302_BTR25 LQRVGVFVDYEEGLVSFYDVESSSHIYSYTGQTFTDKLYPYFSPGLNHGGKNSNPLIITP

B302_BTR18 LQRVGVFVDYEEGLVSFYDVESSSHIYSFTGQTFTDKLYPYFSPAFNNNGKNSAPLIITP

B302_BTR22 PQRVGVFVDYEEGLVSFYDVESSSHIYSYTGQTFTDKLYPYFSPEDNDDGKNSAPLIITP

B302_BTR20 LQRVGVFVDYEEGLVSFYDVESSSHIYSFTGQTFTDKLYPYFSPEDNDDGKNSAPLIITP

B302_BTR19 LQRVGVFVDYEEGLVCFYDVESSSHVYSYTDQTFTDKLFPYFGPCLNDGGKNSTPLIITS

B302_BTR29 PQRVGVFVDYEEGLVCFYDVESSSHIYSYTDQCFSEKLHPCFSPGFNFEGKNSTPLIITH

B302_BTR21 PQRVGVFVDYEEGLVCFYDAESGSHIFSFTGQTFTEKLYPFFNPCGHYAGKNSAPLIITP

B302_BTR31 PEKVGVFVDYEKGLVSFYDVNDGSHIYSFTAQTFTETLYPYFSPGLNDDGKNSKPLIITP

B302_BTR32 PEKVGVFVDYEEGLVSFYNVNDGSHIYSFTAQTFTETLYPYFSPCLNDDGKNSKPLIITP

B302_BTR15 PQVVGVFVDYEEGLVSFYDVESGCHIYSFTGQTFTEKLFPYFSPGNAHKGKNAAPMIISH

B302_BTR17 PQVVGVFVDYEEGLVTFYDVESGSFIYSFTGQTFSEKLSPYFSPYQNNGGRNTAPLIISH

B302_BTR16 PQVVGVFVDYEEGLVSFYDVESSSLIYSFTGQTFSEKVFPYFSPGTTNEGKNAAPLIILN

B302_BTR06 PQKVGVFVDYDDGLVSFYDVESRSHIYSFTGQSFTEKLYPLFSPCINDEGKNSAPLIISA

B302_BTR07 PQKVGVFVDYEDGLVSFYDVESRSHIYSFTGQSFINRLYPYFCPFPNDICKNSASMNISP

B302_BTR05 PQKVGVFVDYEDGLVCFFDVVSRSHIFSFTRQSFINRVYPYFCACFTSNGKHSVPLIISP

B302_BTR04PS PQKVGVFVDYEEGLVSFYDVESRSHIYSFIGQSFTEKVYPFFSPEVIEGGQNP-PLIISP

B302_BTR08 PQTVGVFVDYEEGLVSFYDVESMSHIYSFTGQSFTEKLYPYFSPGFNNGDQNSDPLIISP

B302_BTR09 PQTVGVFVDYEEGLVSFYDVKSRSHIYSFTGQSFTEKLYPFLSPLSNNKGQNSAPLIISP

B302_BTR33 PQTIGIYVDFENGRVSFYDTCACGHIYSFTGQRFTESLLAYFNPDMNDTGNNNAPLVIQP

B302_BTR12 PQKLGIFLDYEAGQVSFYDVKTHTHLYTFI-DAFTESVYPIFSPCLNQDGKNPGPLVITA

B302_BTR13 LKRVGVYVDYEEGQVSFYDVQNKSHIYTFM-DTFKEKLFPFFYLYCCD--KASDTMIICP

B302_BTR30 LRRVGVYLDCEEGQVIFYNAKSGAELYCFS-GPLSEKMLPLFG-----TADKEVPMVLCS

. : : :* : * : *: :. : : : : . : .: :

B302_BTR01 PF--------------

B302_BTR11 VL--------------

B302_BTR02 SC--------------

B302_BTR24 V---------------

B302_BTY VNYNK-----------

B302_BTR23 LG--------------

B302_BTR25 VS--------------

B302_BTR18 V---------------

B302_BTR22 V---------------

B302_BTR20 V---------------

B302_BTR19 LHFLKWMQQYAVDVTL

B302_BTR29 L---------------

B302_BTR21 V---------------

B302_BTR31 V---------------

B302_BTR32 V---------------

B302_BTR15 I---------------

B302_BTR17 V---------------

B302_BTR16 V---------------

B302_BTR06 VK--------------

B302_BTR07 V---------------

B302_BTR05 VNYS------------

B302_BTR04PS VI--------------

B302_BTR08 P---------------

B302_BTR09 V---------------

B302_BTR33 V---------------

B302_BTR12 V---------------

B302_BTR13 VQET------------

B302_BTR30 AADPLL----------

Multiple alignment of TRIM35 B30.2 domains

TRIM35

.

Multiple alignment of B302 domains used for computing the Shannon entropy profile of Trim35 B30.2 domains.

CLUSTAL W (1.83) multiple sequence alignment

TRIM35_01 SDPVILNPNTSAPQLSVSDDLTSVTSST-HRQNQTSDLPLH--RSRVVLGSVGYSDGDHT

TRIM35_02 SDPVILNPNTSAPQLSVSDDLTSVTSST-HRQNQTSDLPLH--RSRVVLGSVGYSDGDHT

TRIM35_03 SAPIILDPNVAHRNLFLSDDLTSLKWS--LNSQAFPDNPERFDEYSCVLGSEGFTSGKHC

TRIM35_04 AAPVVLDPNTAHPDLLVSADLSSVRWS--WSKRALPDNAERFDHHPCVLASEGVRSGTLS

TRIM35_05 SAPIILDPNVAHRNLFLSDDLTSLKWS--LNSQAFPDNPERFDEYSCVLGSEGFTSGKHC

TRIM35_06 YTPVILDPNTAHPHLILSDDLTSVRES--EDKRQFPNNPERFDRFPCVLGSEGFRSGKHS

TRIM35_08 SAPVILDPNTANPHLILSDDLTSGRGT--GNRQPLPPNPERFDWYFCVLGSEGFNSGKHS

TRIM35_09_ SAPVILDPNTANPHLVLSDDLTSLRDSF-LNSKPLPDNPERFDYYSCVLGSEGLNSGKHR

TRIM35_10 SAPVILDPNTANSSLVLSDDLTSMARD--IIIELRPHNPERFDSYCCVLGSEGFTSGTHY

TRIM35_11 PAPVILDPNTANPRLLLSDDLTSVKSLEFSEKQLLPNNPERFDWFFSVLGSEGFNSGTHC

TRIM35_12 SFICLSAFNTAYPCLSLSKDLTSVSNT--GTMQKLPDNPERFDHLVFVLGSKGFSSGCHS

TRIM35_13 AAPVTLDPNTAASCFLLSEDLTTLQCCS-QTF-KLPENPERFNVGAEVLGYEGFSSGRHS

TRIM35_14 TAPVILDPNTASSKQWVSADCTSVQYVL-KKV-HMCDNPKRLFLG--VLASQGFNSGLHC

TRIM35_16 PSAITFNSKTANPWLSLTSSLTCVRYQT--FNSSVQDNPQRFNAALSLMGGQGFTKGRHY

TRIM35_17 HCALTFDPRTANAHLRLSQSNRRAEHLV-SGPRPVPADESRFDHTWQVLCFQGFTSGQHY

TRIM35_18 PAPVILNPNTADVCVSLSDDLTTIRYSE-EEQ-LLPENPERFSFYECVLGSEGLVSGRHS

TRIM35_19 SAPVILDPNTANPRLVLSDDLTSVRYS--RNNLPVPDNPERFDFYSCVLGSEGFTSGKHC

TRIM35_20P SAPVILDPNTANPRLVLSDDLTSVRFN--NKNQPVPDNPERFNYYWCVLASEGFTSGKHC

TRIM35_21 SAPVILDSNTAHPRLVLSDDLTSMRYS--GKDQPVPDNPERFDCYYCVLGSEGFTSGKHC

TRIM35_22 SAPVILDPNTAHPYLELSDDLTSMRYS--GKDQPVPDNPERFDSYPCVLGSVGFTSGKHC

TRIM35_23 SAPVILDPNTANPFLNMSDDLTSVRDR--DDIEPLPDNPERFDQMYCVLGSEGFTSGKHC

TRIM35_24 SAPVILDSNTAHPRLVLSDDLTSVKYS--GKDQPVPDNPERFDCYSCILGSEGFTSGKHC

TRIM35_25 YTPVILDPNTAHPGLILSDDLTTLRNR--RNTQPSPDNPERFDQMYCVLGSEGFISGKHC

TRIM35_26 SAPVILDPNTANPRLVLSDDLTTVRFT--GKDQPVPDNPERFDYYECVLGSEGFTSGKHC

TRIM35_27 AAPVVLDPNSAHPCVRVCAGLRSLRFSR-RCPPALCIAGGR-EGYSSVLGSVALGSGSHR

TRIM35_28 AVPVILDPNTASPWLSISPDFSSMQES--RERQSFPDNPERFDPCVFVLGSEGFSSGRHR

TRIM35_30 TDPVTFDPNTGHPYLLVSDDLTTITYSD-EHFQQLPANPERYDGYTSILGSEGFTAGSHT

TRIM35_31 LAPVILDPNTANSHIVLSEDLTSVRES--ENKQWLPDNPERFD-YPCVLGSVGFCSGKHR

TRIM35_32 SAPVILDSNTAHRWLCPSDDLTRIMFR--G-HMVVPDNPERFDHYYCVLGSEGFNSGKHT

TRIM35_34 SAPVILDPNTANPRLRLSDDLTRVTIR--R-KQPVLDNPERFDCYRCVLGSEGFNSGKHC

TRIM35_35 SAPVILDPNTAYPELYLSDDLTSVTRR--GNRQPVPDNPERFDCCFCVLGSEGFNSGKHC

TRIM35_36 SAPVILDPNTALPELELSDDLTRVTYR--GIKQPVPDNPERFDRCLCVLGSEGFNSGKHC

TRIM35_37 YTPVILDPNTAYPQLYLSDDLTRVTYR--GIKQPVADNPERFDRYSCVLGSEGFNSGKHC

TRIM35_38 SAPVILDPNIAHPQLHLSDDLTRVTIR--EIKQPVPDNPERFDCYLCVLGSEGFNSEKHC

TRIM35_39 PAPVILDPNTAHPDLLLSDDLTSLRCSTESGD--LPDNPERFDEHSCVLGSEGFNSGTHC

TRIM35_41 SAPVILDPNTAHPRLVLSDDLTSVKYS--GKDQPVPDNPERFDSYYCVLGSEGLNSGKHC

TRIM35_42 TAPVILDPNTAHPRLIVSDDLSTVSYS--WDEQPLPDNPERFDIYECVLGSVGFKTGNCS

. . . : :: .

TRIM35_01 WEIEVGK-SRHWSLGVCLELKGKPIT-QPLIPANGFWGLKREGYMYHLM-----TAEACE

TRIM35_02 WEIEVGK-SRHWSLGVCLELKGNPIM-QPLIPANGFWGLKRERYMYHLM-----TAEGCE

TRIM35_03 WDVEVKE-SLYWSVGVTTASNQRKGC-VFFNSD--VWWVQYG------------LDDRFG

TRIM35_04 WAVEVRQ-SSDWTLGVTTELNQRKGW-DFFSAD--VWSVCYDEYS-------VSERPVFG

TRIM35_05 WDVEVKE-SLYWSVGVTTASNQRKGC-VFFNSD--VWWVQYG------------LDDRFG

TRIM35_06 WVVEVSY-SFYWNLGVTTASNQRKGR-DFYDTN--VWSVEYYENPPQDS---LNSTVSTC

TRIM35_08 WDVEVKD-NSGWGLGVTTAINHCKGR-VFYNTG--VWCVWYHS---------DEQTKWAG

TRIM35_09_ WDVEVKK-CSIWSLGLTTASNQRKGR-DFFNTG--VWCVSFG------------LYEPSG

TRIM35_10 WDVEVNE-SPGWSLGVTTASKQRKGR-VFFNTD--FWGVQCG------------PFQLPG

TRIM35_11 WDVEVKG-SSGWSIGVTTASHERTGG-DIFATD--IWNVKYNESVLYEEFYYQFPKPNNE

TRIM35_12 WEVEVGK-NNDWVIGVVKASVARKGK-ISGCPEGGFWTIALSDGQYTAM-----TTPRTQ

TRIM35_13 WDVEVKN-NTYWVIGVASASISRKGK-HVLTPAEGFWTIRLRNGEYKAC-----TAPWSP

TRIM35_14 WDVEVRD-NNHWTLGVVGETVYRKRL-YRMDPKSRFWCFRFVDGKFKKG-----NNPGKA

TRIM35_16 WEVEVYS-STVWTVGVARESVTRKGV-INTMPANGFWTLSLSYGVQYMAG----TSPPTL

TRIM35_17 WELEVSKPWAYIGVTYPNIPRKQKGKRCMVGMNDLSWSLQLDERQMSAWHAG-CKETVAG

TRIM35_18 WDVEVGD-CSEWALGVVKESVQRKEW-FPPSPERGMWTVGLYGGEYRAR-----TPTSAP

TRIM35_19 WVVEVKE-SPCWILGVSTASNQRKGN-VFFSTG--VWSVRYN------------TAISSG

TRIM35_20P WDVEVKKYCYWWSLGVTTASKDRKRS-DFFNTG--VWSVQCG------------TTVGSG

TRIM35_21 WDVEVKE-SEYWNLGVTTASNQWTGR-VFYNTG--VWSVKYK------------QSAGSG

TRIM35_22 WNVEVKE-ISGWSLGVTTASKQKKGS-DFFNTC--VWGVRYN------------TAISSG

TRIM35_23 WDVEVKG-SQFWSLGVTTASNQRKGK-KFFNTG--VWCVRYG------------CTVSTG

TRIM35_24 WDVEVKE-TLYWSLGVTTASNQIKGG-DFFTTG--IWSLGYG------------CTVRSG

TRIM35_25 WDVEVKE-SLYWNFGVTTASNQRKG---FFSTG--VWSVRCE------------LSFGSG

TRIM35_26 WDVEVKE-SRCWSLGVTTASNQRRGW-VFFRTG--VWGVQYG------------CTVRSD

TRIM35_27 WDVHVGD-SSVWALGVISESALQTQD-QLPESGLWILGFQSGEYGQGCC-----GESLSR

TRIM35_28 WEVRVAD-HPKWILGVCKESVVRKRK-FTVTTTAGVWTIGLSKGVYNAL-----TSPRTV

TRIM35_30 WDVEVGD-NTAWAIGVITESTYKNRL-NHFRMGMWYVGFCNGKYGKGYS-----PEAITL

TRIM35_31 WDVEVKE-SRCWSVGVTTASNQRKRD-VFFNTD--VWSVWHD---PYGL---IKSSSNE-

TRIM35_32 WEMEVKK-GGLWSVGVTTASNQRKKL-VFYDSN--VWCVWHDA---------DQYYEESG

TRIM35_34 WIVEVKK-SKIWSLGVTTASNQRKGG-GFFNSD--VWSVWYD----------PDKLFESG

TRIM35_35 WIVEVKE-SKRWSLGVTTASNQRKGE-VFFKSD--VWSVCDG------------WSRGSG

TRIM35_36 WIVELKE-SQYWSLGVTTASNQRKGV-VFFNSD--IWSVSY------------RGSGVSG

TRIM35_37 WIVEVKE-SKRWILGVTTASNQRKGD-VLFKSD--VWSVCYG------------WSGLPG

TRIM35_38 WIVEVKE-SQRWSLGVTTASNQRKGD-VFFNSD--VWCVYYGL---------SAESAESS

TRIM35_39 WDVEVKE-SSSWGLGVTTASNQRKGC-DFFDSG--VWSVQY---RLFEE---------SG

TRIM35_41 WNVEVKE-SQYWSLGVSTASNQRKGS-PFSNTG--VWSVKYK------------QSVGSG

TRIM35_42 WDVEIRG-SECWSLGVTTASNQRKGR-EFCNAD--VWRVTYD---PYNQ---LGSDTS--

* :.: . .

TRIM35_01 INIKRNPEVVRVKLDYVCDDTMEKQRWRRVSFIDASCDAVIERFSRVPLQHKLFPFVIPE

TRIM35_02 INIKRNPEVVRVKLDYVCDDTMEKQRWRRVSFFDASCDAVIERFSRVPLQHKLFPFVIPE

TRIM35_03 FPIKQKLERVRVDLDCDEG---------TVCFSDSGNNTPLYTFTATFTDTVFPFFWS--

TRIM35_04 VRVKQRLERVRVDLDYDGG---------TISFSDAVNNTHIHTFSSNFTHTLIPFFSCYT

TRIM35_05 FPIKQKLERVRVDLDCDEG---------TVCFSDSGNNTPLYTFTATFTDTVFPFFWSN-

TRIM35_06 FTVTQKLECVKVELDCDEG---------TVSFSDPATDTHLHTFTATFTDTVFPFFWGFS

TRIM35_08 FRVKEKLERVRVSLDYDSG---------MVSFSDPVAKTHLHTFTTTFTHSVFPFFNCGL

TRIM35_09_ FIVKQSLERVRVDLDCDRG---------TVSFSDPVTNTHLHTFTTTLTESVFPFF-CSI

TRIM35_10 VQVQKDLECVRVDLDCDSG---------TVSFSDPVTNTHLHTFTPTLTESVFPFF-CSY

TRIM35_11 FIVQRELERVRVNLDYDAG---------TVSFSDPVTKTHLQTFTTTFSDTVLPFLCCYD

TRIM35_12 LKLNSHLERVRVKIDYDAG---------EVSFFDSVDVAPLYTFTDHFTEMMFPFFCPG-

TRIM35_13 LTMTKEPQVVRVVLDMNRS---------RVTFYDLRERTPLFTYTDIITPRAFPYFCSAC

TRIM35_14 IDENKRPNIIRVQLDFEKG---------ELRFIDPFRNKNLCTFTGGFPERVFPYFCSGD

TRIM35_16 LSLEEPLARIGVYLDYKRG---------LVSFYNAESMTHLYTFRDTFTETLFPYFNLGF

TRIM35_17 QLDNTHPLRIGLLLDYEAG---------TLTYYGE-GQVRLHAFHCVFNQALFP------

TRIM35_18 LKLKKKPQKIRVQLDCEAG---------RVTFSDAADNSIIYKYKNKFTEKVFPYFSNTC

TRIM35_19 FTVKQDLECVRVDLDCDRG---------TVSFSDPVTNTHLHTFTTTFTESVFPFFWCGS

TRIM35_20P VTVNQDLERVRVDLDCDRG---------IVSFSDPVTNTHLHTFTTTFTESVFPFFNG--

TRIM35_21 FVVNQDLERVRVDLDCDRG---------TVSFSDPVTNTHLHTYTTTFTESVFPFFYSLG

TRIM35_22 YTVNQDLERVRVDLDCDRG---------TVSFSDPVTNTHLHTFTTTFTESVFPFFYSPI

TRIM35_23 FRVKQDFECVRVDLDCDRG---------TVSFSDPVTNTHLHTFTTTFTESVFPFFYCWS

TRIM35_24 YTVKQELERVRVDLDCDRG---------TVSFSDPLTNTHLHTFTTTFTESVFPFFYCGL

TRIM35_25 FIVKQSLERVRVDLDCDRG---------TVSFSDPLKNTHLHTFTTTFTESVFPYFYFN-

TRIM35_26 FHVNQDLECVRVDLDCDRG---------TVSFSDPVTNTHLHTFTTTFTESVFPFFYSL-

TRIM35_27 LGVRQRVSRVTVLLDWDAG---------KLTFLDSLTGYYISCCSLTFL-----------

TRIM35_28 LNLERRPETVRVKVNMEKG---------EVSFWDTGNNKHLCTFNDKFTGKLFPIFGPG-

TRIM35_30 LRVSQKIKRIRVKLDWDKG---------KVTFIDSDRNISLHVFKHTFTERVFPYF----

TRIM35_31 FHVEQYLECVRVELDYDRG---------TVCFSDPVNNTHLHTFTTTFTDTVFPFLWCDL

TRIM35_32 FRVVQALERVRVDLDYDRG---------TVSFSDPLKSKHIHTYTTTFTDTVYPFFRCY-

TRIM35_34 FHVDQALDRVRVDLDYDRG---------TVSFSDPVNNKHLHTYTTTFTHTLYPFFMCYN

TRIM35_35 FCVLQDLDRVRVDLDYDRG---------TVSFSDPVKNKHLHTYTTTFTHTLYPFFRCF-

TRIM35_36 FRVVQDLDRVLVDLDYDRG---------TVSFSDPVKNKHLHTYTTTFTDTVYPFFCSV-

TRIM35_37 YRVVQALDRVRVDLDYDRG---------TVSFSDPVNNEHLHTYTTTFTDTVYPVFWCG-

TRIM35_38 FRVDQDLDRVRVDLDYDRG---------TVSFSDPVKNKHLHTYTTTFTDTLYPFFCSV-

TRIM35_39 FPVGQKLERVRVQLDYDKG---------RVSFSDPVTNTHIHTFTTTFTETVFPFLYSFT

TRIM35_41 FTVNQDLERVRVDLDCDRG---------TVSFSDPVTNTHLHTYTTTFTESVFPFFWCCS

TRIM35_42 --VEQDLERVRVYLDYDSG---------TVSFSNPVNNRLLHTFTTTFTDTVFPFFC---

: : :: . : : . :

TRIM35_01 DQSIP----LRIA------

TRIM35_02 DQSIP----LRIT------

TRIM35_03 -------------------

TRIM35_04 IF------NLKILPS----

TRIM35_05 -------DFK-ILPVL---

TRIM35_06 --------YQKILPT----

TRIM35_08 SS------FLRILP-----

TRIM35_09_ Y-------QLRILP-----

TRIM35_10 F-------PLRILPLGS--

TRIM35_11 --------SLKILP-----

TRIM35_12 ---------ANINR-----

TRIM35_13 K------------------

TRIM35_14 L------------------

TRIM35_16 LDKVHENEPLKVFMPK---

TRIM35_17 -------------------

TRIM35_18 K------------------

TRIM35_19 LV------YLRIL------

TRIM35_20P SA------SLRILPF----

TRIM35_21 --------SLKILP-----

TRIM35_22 --------ALKILPI----

TRIM35_23 S-------ALRILPIL---

TRIM35_24 FS------SLRIL------

TRIM35_25 -------DIVNCLKIL---

TRIM35_26 MS------SLRILPF----

TRIM35_27 -------------------

TRIM35_28 ---------LQST------

TRIM35_30 -------------------

TRIM35_31 --------FSS--------

TRIM35_32 -------DYIQILPS----

TRIM35_34 NFFSYSSSSLKILPS----

TRIM35_35 -------PSFRILAS----

TRIM35_36 -------SSLQILAS----

TRIM35_37 -------SSLQMLAS----

TRIM35_38 -------SSLQILASSVQD

TRIM35_39 --------YLWIL------

TRIM35_41 PV------SLRILPLSSQ-

TRIM35_42 -------------------

**Multiple alignments used for the paml analysis of btr (first for the entire B30.2 domain, then for each of the segments delimmitted by putative recombination sites).**

Multiple alignment of btr B30.2 domains (nucleotide sequences) with indication of segments 1, 2 and 3 as determined by GARD. The multiple alignments used for PAML analysis of each segment are indicated below.

1. 557

<----Segment1-----------------------------------------

BTR01 GCAATGGACG TGACCCTAGA CAAAGACTCG GCTCACCCCC GTCTGGTCAT

BTR02 TCAGTTGATG TGAATCTGAA TCCTCGGACA GCTCATGCTT ACCTCTACAT

BTR04 GCAGTGGAAT TGACTATGGA TCCCGATACA GCTCATCCAG AACTCCTCCT

BTR05 GCAGTGGATG TGACTCTGGA TCGTGATACC GCTTATCCAG AACTCATCCT

BTR06 GCAGTGGATG TAACTCTGGA TCCTGATACA GCTAATCCAT TTCTCATCCT

BTR07 ACTGTGGATG TGACTCTGGA TCCCGATACA GCTCATCAAA AACTGATTCT

BTR08 ACAGTGAATG TGACTATGGA TCCTGAAACT GCTCACCCCA AACTCTTCTT

BTR09 TCAGTGGATG TGATTCTGGA TCCTGATACA GCTCATCCGA AGCTTATCCT

BTR11 GCAGTGGATG TGACCCTGGA CGCCAACACG GCCCATCCAC GCCTCATCCT

BTR12 GCAAGTGAGC TGATTTTGGA TCCTGCCACG GCTCAGCGTG ATCTCTGTCT

BTR13 TCAGTGGACG TGACCTTTGA CCCGGACACG GCGAACCCTT GGCTCCAGCT

BTR15 GCAGTGGATA TAACTATGGA TCCTGAAACA GCCCATCCCA ATCTAATTCT

BTR16 ACAGTGGACG TGACTCTGGA TCCTGATACA GCTTATTCAA AACTCATCCT

BTR17 GCAGTGGATG TGACTCTGGA TCCTAACACA GCTCATCCAA AACTCATCCT

BTR18 GCAGTGGATG TGACTCTGGA TCCTGATACA GCTAATCCAT ATCTCATCCT

BTR20 GCAGTGGATG TGACTCTGGA TCCTGATACA GCTCATCCTA AACTCATCCT

BTR21 GCAGTGGATG TGACTCTGGA TCCTGATACA GCTCATCCTG AACTCATCCT

BTR22 GCAGTGGATG TGACTCTGGA TCCTGATACA GCTAATCCAT ATCTCATCCT

BTR23 GCAGTGGACG TGACTCTGGA TCCCGATACA GCTCATGTTG GACTCATCCT

BTR24 GCAGTGGATG TGACTCTGGA TCCTGATACA GCTCATCCAG ATCTCATCCT

BTR25 GCAGTGGATG TGACTCTGGA TCCTGATACA GCTCATCCAT ATCTGATCCT

BTR29 GCAGTGGACG TGACTCTGGA TCCTCATACA GCTCATCCTG AACTCATCCT

BTR31 GCAGTGGATG TGACTCTGGA TCATGACACG GCGAATCCAT TCCTCATCCT

BTR32 GCAGTGGATG TGACTCTGGA TCCTGACACG GCGAATCCAT TCCTCATCCT

BTR33 GCAGTGGATT TGACCCTTGA CCACGACACA GCAAACTCCT GGTTGGCGGT

------------------------------------------------------

CTCGGAGGAT GGGAAGCAGG TGCTGTGCAG TGACCGATAC CAGAATGTGC 100

CTCTGAGGAT CGGAAGGAGG TCAGGCATGC AAATAAGCAA CAAGAAGTTC

GTCTGATGAC AGGAAACAAG TGAGAGTTCA AGACGTTGAA CATGAGCTCC

GTCTGATGAT GGAAAAGAAA TGATACAAGG AGACGTTTAT CAAGACCTCC

GACTGATGAC GGGAAACAAG TGACACATGG AGACACTAAC CAAAATCTCC

CTCTGATGAC GGGAAGCAAG TGAGCACTGA GGACACTAAA CAAAAGGTCC

GTCTGAGGAT GACAAACAAG CTGAATTTGG AGAAACACGT CAGCCTGTCC

GTCTGAAGAT GGAAAGCAAG TGCGGTATGG AAACATAAAG CATGACCGCC

ATCTGAAAAC AAGAAGATGG TGTGGTGCAG TGAAAATCAG CAGCATGTTA

GTCTGAAGAT GGGAAGCAAG TGCGCTATGA AGA-GCAGCG TAAAAACTCC

GTCGGAGGAT GGTCATCAGA TTCGTCACCT GGGCTCGTGG CAGGACCTAC

CTCTGATGAT GGAAAACAAG TGACAAATGG TGACATCAAG CTTGAACTCC

GTCTGATGAT AGAAAACAAG TGACTTACGG AGCCACTGTG CAGAAACTCC

GTCTGATGAT GGAAAACAAG TGACCTTTGG AGACAAAGAA CTTAAACTCT

GTCTGATGAT GGAAAACAAG TGAAACATGG AGACATTAAG CAGAAACTCC

GTCTGATGAT GGAAAACAAG TAAAACATGG AGACATCAGA CAGAAACTCC

GTCTGATGAT GGAAAACAAG TGAGAGATGG AGACATTATA CAGAAACTCC

GTCTGATGAT GGAAAACAAG TGAGATGTGG AGACATTGAG CAGGAACTGC

GTCTAAAGAT GAAAAGCAAG TGAGACTTGG AGAAATCATA CAGAAACTCC

GTCTGATGAT GGAAAACGAG TGAGAGATGG AGACATCAGA CAGAAACTCC

GTCTGATGAT GGAAAACAAG TGAGAGATGG AGACATTGAG CAGAAACTCC

GTCTGATGAT GGAAAGCAAG TGAGCTGTGG AGATATCTGG CAGAAACTAC

GTCTGACGAT GAAAAACAAG TGAGTTTGGG ACACATCGAG AGGAACGTTC

GTCTGATGAT GAAAAACAAG TCAGTCATGG AGACATCGAG TATGATGTCC

GTCTGAGGAC AGGAAAAGTG TCAGTGATGG CAACGTAGAA CGCAACTTTC

------------------------------------------------------

-------CAG ACACACTCGA GCGATTCGAC CGTGTGGTGT GTGTTCTGGG 150

-------CAG AAAACCCCAA GAGGTTTGAC CGGGTGATCA ATGTCATGTC

-------CAG ATATCCCAGA GAGGTTTGAT TATTGTCCTG ATGTCCTGGC

-------CAG ATATCCCAGA GAGGTTTGAT CATTGCCCTT CGGTCCTAGG

-------CAA ACAACCCACA GAGATATGAT CAATGCAACA CTGTCCTTGG

-------CAA ACAACCTAGA AAGGTTTGAT AAGTGTATCT GTGTTCTGGG

-------CAG ACAATCCGTG GAGGTTTAAT AAGTGTCCCA GTGTCCTAGG

-------CAG GCAGTGACAA GAGGTTTGAA AACTATATTG TTGTGTTGGG

-------CCA ACCACCGTGA GCGCTTTGAC CGTGTGGTCT GTGTGCTCGG

AGCAACTCCG ACACACCGCG GCGCTTCAGT CCAGCTCTGT TCGTTCTGGC

-------GGG ATGCTCCGGA GCGGTTCGAT ACGGTGGTGA TCGCTCTGGG

-------CAG ACAACCCAGA GCGGTTTAGC ACTTGTTGCT GTGTCCTGGC

-------CAG CCAACCCAAA GAGGTTTGAC AGTTGTTGCT CCGTCCTGGC

-------TAA ACAACCCAGA GAGGTTTGAT TGTTGTCCAT GTGTCCTGGC

-------CAG ACACACCACA GAGATTTGAC AGATATGAAG ATGTCCTGGG

-------CAA ACAAACCACA GAGATTTGAT AGATATGCAA TGGTTCTGGG

-------CAA ACAATCCACA GAGATTTGAT TACTCTGTCT CTGTTCTGGG

-------CAG ACAAACCACA GAGATTTGAT AAATATGAAG ATGTCCTGGG

-------CAG ACAAATCACA GAGATTTGAA AAATGTATCT GCGTCCTGGG

-------CAG ACAAACCACA GAGATTTGAT TACTGTGTCT GTGTCCTGGG

-------CAG ACAAACCACA GAGATTTGAT AGATGTCCCT GTGTCCTGGG

-------CCA ACAAACTAGA GAGATTCAAT AGATATCTAT CTGTTCTGGG

-------CAG AAAACCCAGA GCGGTTTAAT CACACTGTTA GTGTTCTGGG

-------CAG AAATCCCAGA AAGATTCGAT TACACTGTTA GTGTTTTAGG

-------AGA ACAACACTCA ACGTTTTGAC ACGGCACCAT GCATCCTATC

------------------------------------------------------

CCGCCAAGGT ATCAACTCCG GCTGCCATTA CTGGGAGGTC CTGGTGAGTG 200

CAAGGAAGCT TTTCGCTATG GCAGACACCT TTGGGAAGTG GATGTTGGAG

AAAGGAGGGA TTCTCTGCGG GGAGATTTTA CTTCGAGGTT CAGGTGAAGG

AAGAGAGGGA TTCTCCTCAG GGAGATTTTA TTTTGAGGTG AAGGTGAAGG

AAAAGAGGGA TTCTCCTCGA AGAGATTTTA TTTTGAGGTG CAGGTGAAAG

AAAAGAGGGA TTCCTGACGG GGAGATTGTA TTTTGAGGTT CAGATGAAGG

GAAGGAGGGA TTCTGTTCTG GCAAGTTTTA TTTTGAGGTG CAGGTAAAAG

AAAAGAGGGC TTTTCCTCAG GGAGATTTTA TTTTGAAGTA AATGTAAGTG

TCGCGAGGGC TTTAACACTG GACGACACTA CTGGGAGGTA AAGGTAAACG

TCGTGAGGGT TTCTCCTCCG GCAGACACTA CTGGGAGGTG GATGTTGGAC

CCGGCAGGGC TTCTGTTCTG GCCGCCGCTA CTGGGAGGTT CAGGTGGGCG

CAAAGAGGGA TTCAACTCGG GGAGATTTTA TTTTGAAGTG CAGGTAAAAC

AAAAGAGGGA TTCAACTCTG GGAAATTTTA TTTTGAGGTG CAGGTGAAAG

AAAAGAGAGA TTCAGCTCAG GGAGATTTTA TTTTGAGGTG CAGGTAAAAG

AAAGGAGGGA TTCTCCTCAG GGAGATTTTA TTTTGATGTT CAGGTGAAGG

AAAGGAGGGA TTCTCCTCAG GGAGATTTTA TTTTGAGGTG CAGGTGAAGG

AAAGGAGGGA TTCTCCTCAG GGAGGTTTTA TTATGAGGTG CAGGTGAAGG

AAAGGAGGGA TTCTCCTCAG GGAGATTTTA TTATGAGGTG CAGGTGAAGA

AAAGGAGGGA TTCTCCTCGG GGAGATTTTA TTTTGAGGTG CAGGTGAAGG

AAAGGAGGGA TTCTCCTCAG GGAGATTTTA TTTTGAGGTG CAGGTGAAGG

AAAGGAGGGA TTCTCCTCAG GGGGATTTTA TTTTGAGGTT CAGGTGAAGG

AAGGGAGGGA TTCTCCTCGG GGAGGTTTTA TTTTGAGGTG CTGGTGAAAG

GAAACAGGGC TTCAGTTCTG GGAAGTTTTA CTATGAGGTG CAGGTGAAGG

GAAACAGGGA TTCAATTCTG GGAAATTTTA CTATGAGGTG CAGGTGAAGG

CAAAGAGCCT ATCTCAAGGG GAAGGAGTTA CTGGGAAGTA GGAGTCTCAG

------------------------------------------------------

ACAAGACGGA CTGGGATTTG GGCATTGCTG CTCGCACCAT CAACAGGAAA 250

ATAAGACTGA CTGGGATTTG GGGGTAGCCA AACAATCTGT CAACAGGAAA

GAAAGACTGA CTGGGTTGTT GGAGTGGCCA GAGAATCGAT TAACAGGAAG

GCAAGACTGA GTGGATTTTA GGTGTGGTCA GAGAATCAAT TAACAGGAAG

AAAAGACTAA ATGGGATTTA GGAGTGGCCA GTGAATCTGT TAAGAGGAAG

GAAAGACGGA GTGGACTTTA GGAGTGGCCA GAGAATCGAT TAACAGGAAG

GGAAAACGGA GTGGGATTTA GGAGTGGCGA GAGAGTCTGT TAACAGGAAG

GAAAGACTGA ATGGCTTTTG GGTGTGGCTA GAGAATCCCT CAACAGAAAG

GAAAGACCGA CTGGGATCTG GGGGTCGCAA GTCATTCCTG CAACAGAAAG

ACAAGACAGC CTGGACCGTG GGGTTGGCCC GAAGCTCGGC GAGACGCAAG

AGAAGGACGA CTGGTACCTG GGAGTTGCCC GCGGGTCTGT CAACAGAAAG

AAAAGACTGA CTGGGATTTA GGAGTGGTCA GAGGATCAGC TAACAGGAAG

GAAAGACTGA CTGGGATTTA GGAGTAGCCA GAGAATCAGT TAACAGGAAG

AAAAGACTGA TTGGGATTTA GGAGTGGTCA GAGAATCCAT TAACCGGAAG

GAAAGACTGA CTGGAGTTTA GGAGTGGTCA AAGAATCTGT AGACAGGAAG

GAAAGACTAA ATGGGATTTA GGAGTGGTCA GAGAATCTGT AGACAGGAAG

GAAAGATTGA ATGGGAATTA GGAGTGGCCA GAGAATCTGT AGGCAGGAAG

AAAAGACTGA CTGGACTTTA GGAGTGGTCA GAGAATCTGT AGACAGGAAG

GAAAGACTAA ATGGGATCTA GGAGTGGCCA GAGAATCTGT AGACAGAAAA

GAAAGACTGA CTGGGATTTA GGAGTGGTCA GAGAATCCAT TAACAGGAAG

GAAAGACTGA CTGGACTTTA GGAGTGGTCA GAGAATCCAT TAACAGGAAG

GAAAGACGGA CTGGACTTTA GGGGTGGCCA GAGAATCGGT TGACAGAAAA

GGAAGACTGA CTGGACTGTA GGACTGGCCA GAGAATCTAT TAACAGAAAA

GAAAGAAGGA ATGGGATTTA GGAGTGGCCA GAGAGTCCAT CAGCAGGAAA

GCAAAACAGC ATGGGATTTG GGAGTGGCCA GGAAATCAGT CAACAGAAAG

Segment1--------> <----Segment2-

G-GCAAAATA GCCGCCAACC CTGCTAATGG ATTCTGGTTC TTAAGTCTAC 300

G-GAAAATTC ACAATATGTC CATCCAATGG TTTCTGGACG CTCAGTTTAA

G-GAGAAATC ACAGTAAATC CTCAAAATGG ATTCTGGGCT GTGGGTCTGA

G-GGAAAATC AAACTGAGTC CTCAAGACGG ACACTGGTGT GTGGCTCTGA

G-GAAAGATC ACACTCAGTC CTCAGCACGG ATACTGGGCT GTGGGTCTGA

G-GGAAAATC ACAGTGGGTC TTCAGGATGG ATACTGGGCT GTGGGTCTGA

G-GAATAATT ACTCTGAGTC CCAGGAATGG ACTGTGGACT CTGTGGTTGA

G-GGGAGTTC TTCCTCAGTC CTAACGATGG GAATTGGTCT CTGTGGCTGA

G-GTAAAATC AAAGTCAGTC CCAGCCATGG ATACTGGTTT CTTAGCTTGC

G-GAGAAATC CGACTCAACC CTGAAGGTGG ATTCTGGTGT TTGTGGCTGA

G-GCAGGATC TCAGTGAGTA CAACTCAGGG TTACTGGGCT CTAGCCATGA

G-GAAAGATC ACAGAGGCTC CTGAGGATGG ATACTGGGCT GTAGCTTTTA

G-GAACGATC ACAGGGAGTC CTGTGAATGG ATACTGGATT ATAGTTCTGA

G-GAGATATC ACAGCTGCAC CTGAGGCTGG ATACTGGATT ATAATGCTGA

G-GAGAGTTC GCACTGAGTC CCGGTAATAG ATACTGGACT GTGTGGTTGA

G-GAAAGATC ACACTGAGAC CCAGTGATGG ATTCTGGACT GTGTGTTTGA

G-GAACGTAC ACACTCAATC CTAGTAATGG ACACTGGACT GTGGTTCTGA

G-GAGAGATC ACACTGAGTC CCGGTAATGG ATACTGGACT GTGTGGTTGA

G-GAGAGATC AGACTTTGTC CCAGTAATGG ATTCTGGACT GTGTGGTTGA

G-GACAGATC ACAGCGAGTG CCAGTAAAGG ATTCTGGACT GTGGTTCTGA

G-GAGAGATC ACACTGACTC CCAGTAATGG ATTCTGGACT GTGTGGTTGA

G-GAGACATC AGAGTGAGTC CTGAAACTGG AAGCTGGACT GTGGCTCTGA

G-GCCAGATC ACACCGAGTC CTGAGAAAGG GTTCTGGACT GTGTGGCTGA

GAGACAAACC A-GCTGACTC CAGCAAATGG ATTCTGGACC ATGGCTTTGA

G-GTTTGGTG ACTCTGAGTC CTGAGGATGG TTACTGGGCG GTTTGTCTGA

------------------------------------------------------

GTGACAAGCA AGATTATGTC TTCCGCACAG AGCCATCGAC ACCTATAATC 350

AGAATGGCTC TCAGTATGTC GCAAACACAT ACCC-TCCGA CTTCCTTCAA

GGAATGAGAG TGAATATAAA GCCTGTACTG GTCCAGCTGT CTCCCTCTCT

TGAAAGGAGA TCAATACTGG GCCTGTGCTG ATCCAGCGGT C------TCT

GGAATTCTGA TGTGTATTGG GCTTTTGATG CTCCAGCTGT CCGTCTGTCT

GGAATGAGAA TGAATATTGG GCTTATGCTG CTCCAGCTGT CCGTCTTTCT

GGAATGGAGC TGAGTATAAG GCCTGTGACT GTCTGTCTGT GTCTCTCTGC

AAGATGAAAA TAAGTGTGAG GCTTGCGAGT CTCTAACTCT CTCCCTCTCC

GGGACAAGAA CAACTACGCT TTCAGAACTG AGCCTCCCAC TGTGCTTCAT

AAAACGGAGA AGTG-AAAGC GCTGACGGGA AGCAGAGTGG CGCTC--CAT

AGAAGGGCCA GGAGTACAGG GTCTCGTCCT CTCCGCCACT GCTGGTGTCC

GGAAAGGAAA TCAATATCAG GTGTTTAAGT CTCCCACTGT CTCTCTGTCT

GGAATGGAAG CCAGTACAAG GCTCGTGAGT CTCCAACTGT TTCTCTGTCT

GGAATGAGAA TCAGTATCTT GCAATTGATT CTTCTTCTGT CTCTCTGTCT

GGAATGGGAA TGGATATGAA GCCTGTGCTG ATTCTCCTGT CTCTCTGTCT

GGAATGGGAA TCAATATAAA GCCGGTGCTG ATTCTCCTGT CTCTCTGTCT

GGAATGGGAA TGAATATAGA GCCTGTACTG GTCCCTCTGT CTCTCTGTCT

GGAATGGGAA TGAATATAAA TTCTGTGCTG ATTCTCCTGT CTCTCTGTCT

GGAA---GAA TGAATACAAG GCTCTCGCTA GTCCTTCAGT ACCTCTGTCT

GGAATGGGAT TGAATATAAA GCCTGTGCTA GTCCTCCTGT CTCTCTGCCT

GGAA---GAA TGAATACAAG GCTCTCGCTA GTCCTTCAGT ACCTCTGTCT

TTAATGGGAA TGAACTGAGC GCTCGTGCTG ATCCTCCAGT CCTTCTGTCT

GGAATGGAAA TCAGTATGAA GCCCTGGATT CTCCAGCCGT CTCTCTCTCG

TTAATGAGAA TGAATATTTA ATTTGTGATG ATCCAGTGGT CTCTTTCCCT

GAAACGGCTG CGAGTACAGG GCCTGTAACC GTGAATCAGA GCTTCTGTCA

----------------------------------------------------->

GTAAATCCAA AACCGCAGCG GATCACT-GT CAGTGTGGAC TATGAGAGAG 400

CCTCAGTCAC AAGCCTAAAA GGGTGTCCAT TTACCTGGAC TATGATGAGG

CTGAGAGTGA AACCGCAGAA GGTTGGT-GT GTTTGTGGAT TATGAGGAGG

CTAAATGTGA AGCCGCAGAA GGTTGGG-GT GTTTGTGGAT TATGAGGACG

GTAAGAGAAA AACCGCAGAA GGTTGGT-GT GTTTGTGGAT TATGACGACG

GTAAGAGAGA AACCACAGAA GGTCGGT-GT GTTTGTGGAT TATGAAGACG

CTGAAAGTGA AGCCCCAGAC GGTGGGT-GT GTTTGTGGAT TATGAGGAGG

TTGAAAGTGA AGCCCCAGAC AGTGGGT-GT GTTTGTGGAT TATGAGGAAG

CTCAGCAACA AACCTCAAAA AATGGGG-CT ATTTGTGGAC TACGAGAAAG

CTGACCTCGC TCCCCCAGAA ACTCGGC-AT CTTTCTGGAT TATGAAGCCG

GTCGAACATA AACTGAAGAG AGTAGGC-GT CTATGTGGAC TACGAGGAAG

CTGAGAGTGA AGCCTCAGGT TGTGGGT-GT GTTTGTGGAT TATGAAGAGG

CTGAAAGTGA AGCCTCAGGT TGTGGGT-GT GTTTGTGGAT TATGAGGAGG

CTGAGAGTGA AGCCTCAGGT TGTGGGT-GT GTTTGTGGAT TATGAGGAGG

CTGAGAGTGA AGCTGCAGCG GGTCGGT-GT GTTTGTGGAT TATGAGGAGG

CTGAAAGTGA AGCTGCAGCG GGTCGGT-GT GTTTGTGGAT TATGAGGAGG

CTGAAAGTGA AGCCGCAGCG GGTCGGT-GT GTTTGTGGAT TATGAGGAGG

CTGAAAGTGA AGCCGCAGCG GGTCGGT-GT GTTTGTGGAT TATGAGGAGG

GTGAAAGTGA AGCTGCAGCG GGTCGGT-GT GTTTGTGGAT TATGAGGAGG

GTGAAAGTGA AGCCGCAGCG GGTCGGT-GT GTTTGTGGAT TATGAGGAGG

CTGAAAGTGA AGCTGCAGCG GGTCGGT-GT GTTTGTGGAT TATGAGGAGG

CTGAGAGTGA GTCCGCAGCG GGTCGGT-GT GTTTGTGGAT TATGAGGAAG

CTGTCGACGA GCCCTGAGAA GGTTGGC-GT GTTTGTGGAT TATGAGAAGG

CAGAGAGCAA AACCTGAGAA GGTTGGA-GT GTTTGTGGAT TATGAAGAGG

CTAAAGTCTC TTCCACAGAC AATTGGG-AT ATATGTGGAC TTTGAAAATG

<------segment3---------

GCCAGTTGTC TTTTTATAAT GCAGATACAA AGACGTTGAT ATTTACTTAC

GACGCGTATC TTTCTATTGT TCGGATACTG GGACTCATAT CTACTCTTTC

GTTTGGTCTC CTTCTATGAT GTGGAGTCAA GATCTCACAT CTACTCTTTC

GTCTGGTCTG CTTCTTCGAT GTGGTGTCCA GATCTCATAT CTTCTCTTTC

GGCTGGTCTC CTTCTATGAT GTTGAGTCAA GATCTCACAT CTACTCTTTC

GTCTGGTCTC ATTCTATGAT GTGGAGTCCA GATCTCACAT CTACTCTTTC

GTTTAGTCTC ATTTTATGAT GTAGAGTCCA TGTCTCATAT CTACTCGTTC

GTCTGGTCTC CTTTTATGAC GTAAAGTCTA GGTCTCATAT CTACTCTTTC

GCCAAGTCTC CTTTTATGAC GTTGACGCAA AAATGCACAT CCATACGTTC

GTCAGGTGTC CTTCTACGAT GTGAAGACGC ACACTCACCT GTACACATTC

GACAGGTGTC GTTTTATGAC GTGCAGAACA AGAGTCACAT CTACACCTTC

GTTTGGTCTC CTTTTATGAT GTGGAGTCCG GGTGTCATAT TTACTCTTTC

GTTTGGTGTC TTTTTATGAT GTGGAGTCCA GCTCCCTAAT TTATTCATTC

GTTTGGTCAC TTTTTATGAT GTGGAGTCCG GGTCTTTTAT TTATTCTTTC

GTTTGGTCTC CTTTTATGAT GTGGAGTCCA GCTCTCATAT CTACTCTTTC

GTTTGGTCTC CTTTTATGAT GTGGAGTCCA GCTCTCATAT CTACTCTTTC

GTTTGGTCTG CTTTTATGAT GCAGAGTCCG GCTCTCATAT CTTCTCTTTC

GTTTGGTCTC CTTTTATGAT GTGGAGTCCA GCTCTCATAT TTACTCTTAT

GTTTGGTCTC CTTTTATGAT GTGGAGTCCA GCTCTCATAT CTACTCTTTC

GTTTGGTCTT ATTTTATGAT GTGGAGTCCA GCTCTCATAT CTACTCTTTC

GTTTGGTCTC CTTTTATGAT GTGGAGTCCA GCTCTCATAT CTACTCTTAC

GTTTGGTCTG CTTTTATGAT GTGGAGTCCA GCTCTCATAT CTACTCCTAC

GTTTGGTCTC CTTTTATGAT GTGAATGACG GATCTCACAT CTACTCTTTC

GTTTGGTCTC CTTTTATAAT GTGAATGACG GATCTCACAT CTACTCTTTC

GACGGGTTTC TTTTTATGAT ACATGTGCAT GTGGACATAT CTACTCATTC

-----------------------------------------------------

ACAGAC---T CCTTTTCAGA GACTCTATAT CCTTTCTTTA GCCCGTGCAC

A---GAGACA GTTTCACAGA TAAGCTCCAC CCTATCCTCA GCCCAGGACG

ATTGGACAAT CTTTCACTGA GAAAGTCTAT CCTTTCTTCA GCCCTGAGGT

ACTAGACAGT CTTTTATTAA TAGGGTTTAT CCGTATTTTT GTGCGTGTTT

ACTGGACAAT CTTTTACAGA GAAACTCTAT CCTTTATTCA GCCCATGCAT

ACCGGTCAGT CTTTCATCAA TAGACTTTAC CCATATTTTT GCCCCTTTCC

ACTGGTCAGT CTTTCACTGA GAAACTCTAT CCATATTTCA GCCCAGGCTT

ACTGGTCAGT CTTTCACTGA GAAACTCTAT CCATTTTTAA GTCCGTTGAG

ATGGAC---A ACTTTTCGGA GACCATTTAT CCATTCTTCA GTCCCTGTAC

ATCGAT---G CGTTCACTGA GAGCGTTTAT CCCATATTCA GCCCCTGCCT

ATGGAC---A CGTTTAAAGA AAAGCTCTTT CCGTTCTTCT ACCTGT----

ACTGGTCAGA CTTTCACTGA GAAACTTTTC CCATACTTTA GTCCTGGAAA

ACAGGTCAGA CTTTTAGTGA GAAAGTCTTT CCATACTTCA GCCCTGGTAC

ACTGGTCAAA CTTTCTCTGA GAAACTCTCT CCATATTTTA GCCCTTATCA

ACTGGTCAGA CTTTCACTGA TAAACTCTAC CCATATTTCA GCCCAGCCTT

ACTGGTCAGA CTTTCACTGA TAAACTCTAT CCATATTTCA GCCCAGAAGA

ACTGGTCAGA CTTTCACTGA AAAACTCTAC CCATTTTTTA ACCCATGTGG

ACTGGTCAGA CTTTCACTGA TAAACTCTAC CCATATTTCA GCCCAGAAGA

ACTGGTCAGA CTTTCACTGA TAAACTCTAT CCATTTTTTA ACCCATGTAG

ACTAGTCAGA CTTTCACTGA TAAACTCCAC CCATTGTTTA GCCCATGTCC

ACTGGTCAGA CTTTCACTGA TAAACTTTAT CCATATTTCA GCCCAGGTCT

ACTGATCAGT GTTTCAGTGA AAAACTCCAT CCATGTTTTA GTCCAGGGTT

ACTGCTCAGA CTTTCACTGA AACACTCTAT CCATACTTCA GTCCAGGCCT

ACTGCTCAGA CTTTCACTGA AACACTCTAT CCATACTTCA GTCCATGTCT

ACTGGGCAGC GCTTTACTGA AAGCTTACTG GCCTACTTCA ACCCAGACAT

------------------------------------------------------

CAACAAATCT GGCAAAAACG AAGCACCACT TATTATT-TG TCCTCCTTTT

ACCCCATGGA GAGAAGAACA CTGCTCCCCT TATCATCAGC AGCAGCTGCA

TATTGAGGGA GGTCAAAATC C---CCCGCT GATCATC-TC ACCAGTTATT

TACCAGTAAC GGTAAACATT CAGTACCACT GATCATC-TC ACCTGTTAAT

TAATGATGAA GGTAAAAATT CAGCACCGCT GATCATC-TC AGCTGTTAAA

TAATGATATC TGTAAAAATT CAGCATCAAT GAACATC-TC ACCTGTTAA-

TAATAATGGA GATCAAAATT CAGACCCACT GATCATT-TC ACCCCCCCA-

TAATAATAAA GGTCAAAATT CAGCACCACT CATCATC-TC ACCTGTGAC-

CAACAAAAAC AGCAAAAATG AAGCTCCGCT GGTGATC-AC ACCTGTGCTA

CAACCAGGAC GGCAAGAATC CCGGACCGCT GGTCATC-AC CGCCGTCAA-

--ACTGCTGC GATAAAGCCT CCGATACCAT GATCATCTGC CCCGTTCAGG

TGCACACAAA GGTAAAAATG CAGCACCAAT GATTATC-TC ACATATCAG-

TACAAATGAG GGTAAAAATG CTGCACCACT GATCATC-TT AAATGTAAA-

AAATAATGGA GGTAGAAACA CAGCTCCACT GATCATC-TC ACATGTCAG-

TAATAATAAT GGTAAAAACT CAGCTCCTCT AATCATC-AC ACCTGTAAG-

TAATGATGAC GGTAAAAACT CAGCTCCTCT AATCATC-AC ACCTGTAAG-

TCATTATGCA GGTAAAAACT CAGCACCACT GATCATC-AC ACCTGTCAA-

TAATGATGAC GGTAAAAACT CAGCTCCTCT GATCATC-AC ACCTGTCAA-

TAATATTGAT GGTAATAACT CGGATCCTCT AATCATC-AC ACCTCTAGGA

AAACTGTGCA GGTAAAAACT CAAACCCACT GATCATC-AC ACCTGTCTA-

AAACCATGGA GGTAAAAACT CAAACCCACT GATCATC-AC ACCTGTCAGT

TAATTTTGAA GGTAAAAACT CAACCCCACT GATCATC-AC TCATCTCAG-

TAATGATGAC GGGAAGAACT CAAAGCCACT GATCATC-AC ACCTGTCAT-

TAATGATGAC GGGAAGAACT CAAAGCCACT GATCATC-AC ACCTGTCTA-

GAATGACACA GGAAATAACA ATGCCCCGCT GGTTAT--AC AACCTGT-CA

------>

-------

G------

TA-----

TACAGC-

-------

-------

-------

-------

AG-----

-------

AGACGCC

-------

-------

-------

-------

-------

-------

-------

G------

-------

TA-----

-------

-------

-------

G------

Segment 1

25 252

BTR01 GCAATGGACG TGACCCTAGA CAAAGACTCG GCTCACCCCC GTCTGGTCAT CTCGGAGGAT GGGAAGCAGG TGCTGTGCAG TGACCGATAC CAGAATGTGC CAGACACACT CGAGCGATTC GACCGTGTGG TGTGTGTTCT GGGCCGCCAA GGTATCAACT CCGGCTGCCA TTACTGGGAG GTCCTGGTGA GTGACAAGAC GGACTGGGAT TTGGGCATTG CTGCTCGCAC CATCAACAGG AAAGGCAAAA TA

BTR02 TCAGTTGATG TGAATCTGAA TCCTCGGACA GCTCATGCTT ACCTCTACAT CTCTGAGGAT CGGAAGGAGG TCAGGCATGC AAATAAGCAA CAAGAAGTTC CAGAAAACCC CAAGAGGTTT GACCGGGTGA TCAATGTCAT GTCCAAGGAA GCTTTTCGCT ATGGCAGACA CCTTTGGGAA GTGGATGTTG GAGATAAGAC TGACTGGGAT TTGGGGGTAG CCAAACAATC TGTCAACAGG AAAGGAAAAT TC

BTR04 GCAGTGGAAT TGACTATGGA TCCCGATACA GCTCATCCAG AACTCCTCCT GTCTGATGAC AGGAAACAAG TGAGAGTTCA AGACGTTGAA CATGAGCTCC CAGATATCCC AGAGAGGTTT GATTATTGTC CTGATGTCCT GGCAAAGGAG GGATTCTCTG CGGGGAGATT TTACTTCGAG GTTCAGGTGA AGGGAAAGAC TGACTGGGTT GTTGGAGTGG CCAGAGAATC GATTAACAGG AAGGGAGAAA TC

BTR05 GCAGTGGATG TGACTCTGGA TCGTGATACC GCTTATCCAG AACTCATCCT GTCTGATGAT GGAAAAGAAA TGATACAAGG AGACGTTTAT CAAGACCTCC CAGATATCCC AGAGAGGTTT GATCATTGCC CTTCGGTCCT AGGAAGAGAG GGATTCTCCT CAGGGAGATT TTATTTTGAG GTGAAGGTGA AGGGCAAGAC TGAGTGGATT TTAGGTGTGG TCAGAGAATC AATTAACAGG AAGGGGAAAA TC

BTR06 GCAGTGGATG TAACTCTGGA TCCTGATACA GCTAATCCAT TTCTCATCCT GACTGATGAC GGGAAACAAG TGACACATGG AGACACTAAC CAAAATCTCC CAAACAACCC ACAGAGATAT GATCAATGCA ACACTGTCCT TGGAAAAGAG GGATTCTCCT CGAAGAGATT TTATTTTGAG GTGCAGGTGA AAGAAAAGAC TAAATGGGAT TTAGGAGTGG CCAGTGAATC TGTTAAGAGG AAGGGAAAGA TC

BTR07 ACTGTGGATG TGACTCTGGA TCCCGATACA GCTCATCAAA AACTGATTCT CTCTGATGAC GGGAAGCAAG TGAGCACTGA GGACACTAAA CAAAAGGTCC CAAACAACCT AGAAAGGTTT GATAAGTGTA TCTGTGTTCT GGGAAAAGAG GGATTCCTGA CGGGGAGATT GTATTTTGAG GTTCAGATGA AGGGAAAGAC GGAGTGGACT TTAGGAGTGG CCAGAGAATC GATTAACAGG AAGGGGAAAA TC

BTR08 ACAGTGAATG TGACTATGGA TCCTGAAACT GCTCACCCCA AACTCTTCTT GTCTGAGGAT GACAAACAAG CTGAATTTGG AGAAACACGT CAGCCTGTCC CAGACAATCC GTGGAGGTTT AATAAGTGTC CCAGTGTCCT AGGGAAGGAG GGATTCTGTT CTGGCAAGTT TTATTTTGAG GTGCAGGTAA AAGGGAAAAC GGAGTGGGAT TTAGGAGTGG CGAGAGAGTC TGTTAACAGG AAGGGAATAA TT

BTR09 TCAGTGGATG TGATTCTGGA TCCTGATACA GCTCATCCGA AGCTTATCCT GTCTGAAGAT GGAAAGCAAG TGCGGTATGG AAACATAAAG CATGACCGCC CAGGCAGTGA CAAGAGGTTT GAAAACTATA TTGTTGTGTT GGGAAAAGAG GGCTTTTCCT CAGGGAGATT TTATTTTGAA GTAAATGTAA GTGGAAAGAC TGAATGGCTT TTGGGTGTGG CTAGAGAATC CCTCAACAGA AAGGGGGAGT TC

BTR11 GCAGTGGATG TGACCCTGGA CGCCAACACG GCCCATCCAC GCCTCATCCT ATCTGAAAAC AAGAAGATGG TGTGGTGCAG TGAAAATCAG CAGCATGTTA CCAACCACCG TGAGCGCTTT GACCGTGTGG TCTGTGTGCT CGGTCGCGAG GGCTTTAACA CTGGACGACA CTACTGGGAG GTAAAGGTAA ACGGAAAGAC CGACTGGGAT CTGGGGGTCG CAAGTCATTC CTGCAACAGA AAGGGTAAAA TC

BTR12 GCAAGTGAGC TGATTTTGGA TCCTGCCACG GCTCAGCGTG ATCTCTGTCT GTCTGAAGAT GGGAAGCAAG TGCGCTATGA AGAGCAGCGT AAAAACTCCA GCAACTCCGA CACACCGCGG CGCTTCAGTC CAGCTCTGTT CGTTCTGGCT CGTGAGGGTT TCTCCTCCGG CAGACACTAC TGGGAGGTGG ATGTTGGACA CAAGACAGCC TGGACCGTGG GGTTGGCCCG AAGCTCGGCG AGACGCAAGG GA

BTR13 TCAGTGGACG TGACCTTTGA CCCGGACACG GCGAACCCTT GGCTCCAGCT GTCGGAGGAT GGTCATCAGA TTCGTCACCT GGGCTCGTGG CAGGACCTAC GGGATGCTCC GGAGCGGTTC GATACGGTGG TGATCGCTCT GGGCCGGCAG GGCTTCTGTT CTGGCCGCCG CTACTGGGAG GTTCAGGTGG GCGAGAAGGA CGACTGGTAC CTGGGAGTTG CCCGCGGGTC TGTCAACAGA AAGGGCAGGA TC

BTR15 GCAGTGGATA TAACTATGGA TCCTGAAACA GCCCATCCCA ATCTAATTCT CTCTGATGAT GGAAAACAAG TGACAAATGG TGACATCAAG CTTGAACTCC CAGACAACCC AGAGCGGTTT AGCACTTGTT GCTGTGTCCT GGCCAAAGAG GGATTCAACT CGGGGAGATT TTATTTTGAA GTGCAGGTAA AACAAAAGAC TGACTGGGAT TTAGGAGTGG TCAGAGGATC AGCTAACAGG AAGGGAAAGA TC

BTR16 ACAGTGGACG TGACTCTGGA TCCTGATACA GCTTATTCAA AACTCATCCT GTCTGATGAT AGAAAACAAG TGACTTACGG AGCCACTGTG CAGAAACTCC CAGCCAACCC AAAGAGGTTT GACAGTTGTT GCTCCGTCCT GGCAAAAGAG GGATTCAACT CTGGGAAATT TTATTTTGAG GTGCAGGTGA AAGGAAAGAC TGACTGGGAT TTAGGAGTAG CCAGAGAATC AGTTAACAGG AAGGGAACGA TC

BTR17 GCAGTGGATG TGACTCTGGA TCCTAACACA GCTCATCCAA AACTCATCCT GTCTGATGAT GGAAAACAAG TGACCTTTGG AGACAAAGAA CTTAAACTCT TAAACAACCC AGAGAGGTTT GATTGTTGTC CATGTGTCCT GGCAAAAGAG AGATTCAGCT CAGGGAGATT TTATTTTGAG GTGCAGGTAA AAGAAAAGAC TGATTGGGAT TTAGGAGTGG TCAGAGAATC CATTAACCGG AAGGGAGATA TC

BTR18 GCAGTGGATG TGACTCTGGA TCCTGATACA GCTAATCCAT ATCTCATCCT GTCTGATGAT GGAAAACAAG TGAAACATGG AGACATTAAG CAGAAACTCC CAGACACACC ACAGAGATTT GACAGATATG AAGATGTCCT GGGAAAGGAG GGATTCTCCT CAGGGAGATT TTATTTTGAT GTTCAGGTGA AGGGAAAGAC TGACTGGAGT TTAGGAGTGG TCAAAGAATC TGTAGACAGG AAGGGAGAGT TC

BTR20 GCAGTGGATG TGACTCTGGA TCCTGATACA GCTCATCCTA AACTCATCCT GTCTGATGAT GGAAAACAAG TAAAACATGG AGACATCAGA CAGAAACTCC CAAACAAACC ACAGAGATTT GATAGATATG CAATGGTTCT GGGAAAGGAG GGATTCTCCT CAGGGAGATT TTATTTTGAG GTGCAGGTGA AGGGAAAGAC TAAATGGGAT TTAGGAGTGG TCAGAGAATC TGTAGACAGG AAGGGAAAGA TC

BTR21 GCAGTGGATG TGACTCTGGA TCCTGATACA GCTCATCCTG AACTCATCCT GTCTGATGAT GGAAAACAAG TGAGAGATGG AGACATTATA CAGAAACTCC CAAACAATCC ACAGAGATTT GATTACTCTG TCTCTGTTCT GGGAAAGGAG GGATTCTCCT CAGGGAGGTT TTATTATGAG GTGCAGGTGA AGGGAAAGAT TGAATGGGAA TTAGGAGTGG CCAGAGAATC TGTAGGCAGG AAGGGAACGT AC

BTR22 GCAGTGGATG TGACTCTGGA TCCTGATACA GCTAATCCAT ATCTCATCCT GTCTGATGAT GGAAAACAAG TGAGATGTGG AGACATTGAG CAGGAACTGC CAGACAAACC ACAGAGATTT GATAAATATG AAGATGTCCT GGGAAAGGAG GGATTCTCCT CAGGGAGATT TTATTATGAG GTGCAGGTGA AGAAAAAGAC TGACTGGACT TTAGGAGTGG TCAGAGAATC TGTAGACAGG AAGGGAGAGA TC

BTR23 GCAGTGGACG TGACTCTGGA TCCCGATACA GCTCATGTTG GACTCATCCT GTCTAAAGAT GAAAAGCAAG TGAGACTTGG AGAAATCATA CAGAAACTCC CAGACAAATC ACAGAGATTT GAAAAATGTA TCTGCGTCCT GGGAAAGGAG GGATTCTCCT CGGGGAGATT TTATTTTGAG GTGCAGGTGA AGGGAAAGAC TAAATGGGAT CTAGGAGTGG CCAGAGAATC TGTAGACAGA AAAGGAGAGA TC

BTR24 GCAGTGGATG TGACTCTGGA TCCTGATACA GCTCATCCAG ATCTCATCCT GTCTGATGAT GGAAAACGAG TGAGAGATGG AGACATCAGA CAGAAACTCC CAGACAAACC ACAGAGATTT GATTACTGTG TCTGTGTCCT GGGAAAGGAG GGATTCTCCT CAGGGAGATT TTATTTTGAG GTGCAGGTGA AGGGAAAGAC TGACTGGGAT TTAGGAGTGG TCAGAGAATC CATTAACAGG AAGGGACAGA TC

BTR25 GCAGTGGATG TGACTCTGGA TCCTGATACA GCTCATCCAT ATCTGATCCT GTCTGATGAT GGAAAACAAG TGAGAGATGG AGACATTGAG CAGAAACTCC CAGACAAACC ACAGAGATTT GATAGATGTC CCTGTGTCCT GGGAAAGGAG GGATTCTCCT CAGGGGGATT TTATTTTGAG GTTCAGGTGA AGGGAAAGAC TGACTGGACT TTAGGAGTGG TCAGAGAATC CATTAACAGG AAGGGAGAGA TC

BTR29 GCAGTGGACG TGACTCTGGA TCCTCATACA GCTCATCCTG AACTCATCCT GTCTGATGAT GGAAAGCAAG TGAGCTGTGG AGATATCTGG CAGAAACTAC CCAACAAACT AGAGAGATTC AATAGATATC TATCTGTTCT GGGAAGGGAG GGATTCTCCT CGGGGAGGTT TTATTTTGAG GTGCTGGTGA AAGGAAAGAC GGACTGGACT TTAGGGGTGG CCAGAGAATC GGTTGACAGA AAAGGAGACA TC

BTR31 GCAGTGGATG TGACTCTGGA TCATGACACG GCGAATCCAT TCCTCATCCT GTCTGACGAT GAAAAACAAG TGAGTTTGGG ACACATCGAG AGGAACGTTC CAGAAAACCC AGAGCGGTTT AATCACACTG TTAGTGTTCT GGGGAAACAG GGCTTCAGTT CTGGGAAGTT TTACTATGAG GTGCAGGTGA AGGGGAAGAC TGACTGGACT GTAGGACTGG CCAGAGAATC TATTAACAGA AAAGGCCAGA TC

BTR32 GCAGTGGATG TGACTCTGGA TCCTGACACG GCGAATCCAT TCCTCATCCT GTCTGATGAT GAAAAACAAG TCAGTCATGG AGACATCGAG TATGATGTCC CAGAAATCCC AGAAAGATTC GATTACACTG TTAGTGTTTT AGGGAAACAG GGATTCAATT CTGGGAAATT TTACTATGAG GTGCAGGTGA AGGGAAAGAA GGAATGGGAT TTAGGAGTGG CCAGAGAGTC CATCAGCAGG AAAGAGACAA AC

BTR33 GCAGTGGATT TGACCCTTGA CCACGACACA GCAAACTCCT GGTTGGCGGT GTCTGAGGAC AGGAAAAGTG TCAGTGATGG CAACGTAGAA CGCAACTTTC AGAACAACAC TCAACGTTTT GACACGGCAC CATGCATCCT ATCCAAAGAG CCTATCTCAA GGGGAAGGAG TTACTGGGAA GTAGGAGTCT CAGGCAAAAC AGCATGGGAT TTGGGAGTGG CCAGGAAATC AGTCAACAGA AAGGGTTTGG TG

Segment2

25 90

BTR01 TTAAGTCTAC GTGACGATTA TGTCTTCCGC ACAGAGCCAT CGACACCTAT AATCGTAAAT CCGCAGCGGA TCACTGTCAG TGTGGACTAT

BTR02 CTCAGTTTAA AGAATCAGTA TGTCGCAAAC ACATACCCTC CGACTTCCTT CAACCTCAGT CCTAAAAGGG TGTCCATTTA CCTGGACTAT

BTR04 GTGGGTCTGA GGAATGAATA TAAAGCCTGT ACTGGTCCAG CTGTCTCCCT CTCTCTGAGA CCGCAGAAGG TTGGTGTGTT TGTGGATTAT

BTR05 GTGGCTCTGA TGAAACAATA CTGGGCCTGT GCTGATCCAG CGGTCTCTCT AAATGTGAAG CCGCAGAAGG TTGGGGTGTT TGTGGATTAT

BTR06 GTGGGTCTGA GGAATGTGTA TTGGGCTTTT GATGCTCCAG CTGTCCGTCT GTCTGTAAGA CCGCAGAAGG TTGGTGTGTT TGTGGATTAT

BTR07 GTGGGTCTGA GGAATGAATA TTGGGCTTAT GCTGCTCCAG CTGTCCGTCT TTCTGTAAGA CCACAGAAGG TCGGTGTGTT TGTGGATTAT

BTR08 CTGTGGTTGA GGAATGAGTA TAAGGCCTGT GACTGTCTGT CTGTGTCTCT CTGCCTGAAA CCCCAGACGG TGGGTGTGTT TGTGGATTAT

BTR09 CTGTGGCTGA AAGATAAGTG TGAGGCTTGC GAGTCTCTAA CTCTCTCCCT CTCCTTGAAA CCCCAGACAG TGGGTGTGTT TGTGGATTAT

BTR11 CTTAGCTTGC GGGACAACTA CGCTTTCAGA ACTGAGCCTC CCACTGTGCT TCATCTCAGC CCTCAAAAAA TGGGGCTATT TGTGGACTAC

BTR12 TTGTGGCTGA AAAACGAAGT GAAAGCGCTG ACGGGAAGCA GAGTGGCGCT CCATCTGACC CCCCAGAAAC TCGGCATCTT TCTGGATTAT

BTR13 CTAGCCATGA AGAAGGAGTA CAGGGTCTCG TCCTCTCCGC CACTGCTGGT GTCCGTCGAA CTGAAGAGAG TAGGCGTCTA TGTGGACTAC

BTR15 GTAGCTTTTA GGAAACAATA TCAGGTGTTT AAGTCTCCCA CTGTCTCTCT GTCTCTGAGA CCTCAGGTTG TGGGTGTGTT TGTGGATTAT

BTR16 ATAGTTCTGA GGAATCAGTA CAAGGCTCGT GAGTCTCCAA CTGTTTCTCT GTCTCTGAAA CCTCAGGTTG TGGGTGTGTT TGTGGATTAT

BTR17 ATAATGCTGA GGAATCAGTA TCTTGCAATT GATTCTTCTT CTGTCTCTCT GTCTCTGAGA CCTCAGGTTG TGGGTGTGTT TGTGGATTAT

BTR18 GTGTGGTTGA GGAATGGATA TGAAGCCTGT GCTGATTCTC CTGTCTCTCT GTCTCTGAGA CTGCAGCGGG TCGGTGTGTT TGTGGATTAT

BTR20 GTGTGTTTGA GGAATCAATA TAAAGCCGGT GCTGATTCTC CTGTCTCTCT GTCTCTGAAA CTGCAGCGGG TCGGTGTGTT TGTGGATTAT

BTR21 GTGGTTCTGA GGAATGAATA TAGAGCCTGT ACTGGTCCCT CTGTCTCTCT GTCTCTGAAA CCGCAGCGGG TCGGTGTGTT TGTGGATTAT

BTR22 GTGTGGTTGA GGAATGAATA TAAATTCTGT GCTGATTCTC CTGTCTCTCT GTCTCTGAAA CCGCAGCGGG TCGGTGTGTT TGTGGATTAT

BTR23 GTGTGGTTGA GGAAGGAATA CAAGGCTCTC GCTAGTCCTT CAGTACCTCT GTCTGTGAAA CTGCAGCGGG TCGGTGTGTT TGTGGATTAT

BTR24 GTGGTTCTGA GGAATGAATA TAAAGCCTGT GCTAGTCCTC CTGTCTCTCT GCCTGTGAAA CCGCAGCGGG TCGGTGTGTT TGTGGATTAT

BTR25 GTGTGGTTGA GGAAGGAATA CAAGGCTCTC GCTAGTCCTT CAGTACCTCT GTCTCTGAAA CTGCAGCGGG TCGGTGTGTT TGTGGATTAT

BTR29 GTGGCTCTGA TTAATGAACT GAGCGCTCGT GCTGATCCTC CAGTCCTTCT GTCTCTGAGA CCGCAGCGGG TCGGTGTGTT TGTGGATTAT

BTR31 GTGTGGCTGA GGAATCAGTA TGAAGCCCTG GATTCTCCAG CCGTCTCTCT CTCGCTGTCG CCTGAGAAGG TTGGCGTGTT TGTGGATTAT

BTR32 ATGGCTTTGA TTAATGAATA TTTAATTTGT GATGATCCAG TGGTCTCTTT CCCTCAGAGA CCTGAGAAGG TTGGAGTGTT TGTGGATTAT

BTR33 GTTTGTCTGA GAAACGAGTA CAGGGCCTGT AACCGTGAAT CAGAGCTTCT GTCACTAAAG CCACAGACAA TTGGGATATA TGTGGACTTT

Segment3

23 117

BTR01 AAGACGTTGA TATTTACTTA CACAGACTCC TTTTCAGAGA CTCTATATCC TTTCTTTAGC CCGTGCACCA ACAAATCTGG CAAAAACGAA GCACCACTTA TTATTTGTCC TCCTTTT

BTR02 GGGACTCATA TCTACTCTTT CAGAGACAGT TTCACAGATA AGCTCCACCC TATCCTCAGC CCAGGACGAC CCCATGGAGA GAAGAACACT GCTCCCCTTA TCATCAGCAG CAGCTGC

BTR04 AGATCTCACA TCTACTCTTT CATTGGACAA TCTTTCACTG AGAAAGTCTA TCCTTTCTTC AGCCCTGAGG TTATTGAGGG AGGTCAAAAT CCCCCGCTGA TCATCTCACC AGTTATT

BTR05 CATATCTTCT CTTTCACTAG ACAGTCTTTT ATTAATAGGG TTTATCCGTA TTTTTGTGCG TGTTTTACCA GTAACGGTAA ACATTCAGTA CCACTGATCA TCTCACCTGT TAATTAC

BTR06 AGATCTCACA TCTACTCTTT CACTGGACAA TCTTTTACAG AGAAACTCTA TCCTTTATTC AGCCCATGCA TTAATGATGA AGGTAAAAAT TCAGCACCGC TGATCATCTC AGCTGTT

BTR07 AGATCTCACA TCTACTCTTT CACCGGTCAG TCTTTCATCA ATAGACTTTA CCCATATTTT TGCCCCTTTC CTAATGATAT CTGTAAAAAT TCAGCATCAA TGAACATCTC ACCTGTT

BTR08 ATGTCTCATA TCTACTCGTT CACTGGTCAG TCTTTCACTG AGAAACTCTA TCCATATTTC AGCCCAGGCT TTAATAATGG AGATCAAAAT TCAGACCCAC TGATCATTTC ACCCCCC

BTR09 AGGTCTCATA TCTACTCTTT CACTGGTCAG TCTTTCACTG AGAAACTCTA TCCATTTTTA AGTCCGTTGA GTAATAATAA AGGTCAAAAT TCAGCACCAC TCATCATCTC ACCTGTG

BTR11 AAAATGCACA TCCATACGTT CATGGACAAC TTTTCGGAGA CCATTTATCC ATTCTTCAGT CCCTGTACCA ACAAAAACAG CAAAAATGAA GCTCCGCTGG TGATCACACC TGTGCTA

BTR15 GGGTGTCATA TTTACTCTTT CACTGGTCAG ACTTTCACTG AGAAACTTTT CCCATACTTT AGTCCTGGAA ATGCACACAA AGGTAAAAAT GCAGCACCAA TGATTATCTC ACATATC

BTR16 AGCTCCCTAA TTTATTCATT CACAGGTCAG ACTTTTAGTG AGAAAGTCTT TCCATACTTC AGCCCTGGTA CTACAAATGA GGGTAAAAAT GCTGCACCAC TGATCATCTT AAATGTA

BTR17 GGGTCTTTTA TTTATTCTTT CACTGGTCAA ACTTTCTCTG AGAAACTCTC TCCATATTTT AGCCCTTATC AAAATAATGG AGGTAGAAAC ACAGCTCCAC TGATCATCTC ACATGTC

BTR18 AGCTCTCATA TCTACTCTTT CACTGGTCAG ACTTTCACTG ATAAACTCTA CCCATATTTC AGCCCAGCCT TTAATAATAA TGGTAAAAAC TCAGCTCCTC TAATCATCAC ACCTGTA

BTR20 AGCTCTCATA TCTACTCTTT CACTGGTCAG ACTTTCACTG ATAAACTCTA TCCATATTTC AGCCCAGAAG ATAATGATGA CGGTAAAAAC TCAGCTCCTC TAATCATCAC ACCTGTA

BTR21 GGCTCTCATA TCTTCTCTTT CACTGGTCAG ACTTTCACTG AAAAACTCTA CCCATTTTTT AACCCATGTG GTCATTATGC AGGTAAAAAC TCAGCACCAC TGATCATCAC ACCTGTC

BTR22 AGCTCTCATA TTTACTCTTA TACTGGTCAG ACTTTCACTG ATAAACTCTA CCCATATTTC AGCCCAGAAG ATAATGATGA CGGTAAAAAC TCAGCTCCTC TGATCATCAC ACCTGTC

BTR23 TCTCATATCT ACTCTTTCAC TGGTCAGACT TTCACTGATA AACTCTATCC ATTTTTTAAC CCATGTAGTA ATATTGATGG TAATAACTCG GATCCTCTAA TCATCACACC TCTAGGA

BTR24 AGCTCTCATA TCTACTCTTT CACTAGTCAG ACTTTCACTG ATAAACTCCA CCCATTGTTT AGCCCATGTC CAAACTGTGC AGGTAAAAAC TCAAACCCAC TGATCATCAC ACCTGTC

BTR25 TCTCATATCT ACTCTTACAC TGGTCAGACT TTCACTGATA AACTTTATCC ATATTTCAGC CCAGGTCTAA ACCATGGAGG TAAAAACTCA AACCCACTGA TCATCACACC TGTCAGT

BTR29 AGCTCTCATA TCTACTCCTA CACTGATCAG TGTTTCAGTG AAAAACTCCA TCCATGTTTT AGTCCAGGGT TTAATTTTGA AGGTAAAAAC TCAACCCCAC TGATCATCAC TCATCTC

BTR31 GGATCTCACA TCTACTCTTT CACTGCTCAG ACTTTCACTG AAACACTCTA TCCATACTTC AGTCCAGGCC TTAATGATGA CGGGAAGAAC TCAAAGCCAC TGATCATCAC ACCTGTC

BTR32 GGATCTCACA TCTACTCTTT CACTGCTCAG ACTTTCACTG AAACACTCTA TCCATACTTC AGTCCATGTC TTAATGATGA CGGGAAGAAC TCAAAGCCAC TGATCATCAC ACCTGTC

BTR33 TGTGGACATA TCTACTCATT CACTGGGCAG CGCTTTACTG AAAGCTTACT GGCCTACTTC AACCCAGACA TGAATGACAC AGGAAATAAC AATGCCCCGC TGGTTATACA ACCTGTC

Sequences kept in the paml analysis of the segment3 were 50% similar or more to the other sequences of the dataset.
