# Supplementary material for: Origin and Evolution of TRIM Proteins: New Insights from the Complete TRIM Repertoire of Zebrafish and Pufferfish
Source: PLoS One. 2011 Jul 15;6(7):e22022. doi: 10.1371/journal.pone.0022022 (PMC3137616; doi:10.1371/journal.pone.0022022)
Supplement: Figure S6 — A table showing the results if of the likelihood ratio test (LRT) of positive selection of zebrafish trim35/hltr and btr B30.2 domains. (DOC) [file pone.0022022.s006.doc]

FigureS6. Results of LRT for positive selection

| TRIM | Region^1^ | Model^2^ | 2 Δ lnL | *p*-value | | No sites |
| --- | --- | --- | --- | --- | --- | --- |
|  |  |  |  |  | |  |
| TRIM35 | B30.2 complete | PAML M1-M2 | 194.26 | *p* < 0.001 | | 12 |
|  |  | PAML M7-M8 | 180.33 | *p* < 0.001 | | 11 |
|  |  |  |  |  | |  |
| BTR | B30.2 complete | PAML M1-M2 | 39.44 | *p* < 0.001 | | 4 |
|  |  | PAML M7-M8 | 54.48 | *p* < 0.001 | | 5 |
|  |  |  |  |  | |  |
| BTR | B30.1 1to 252 | PAML M1-M2 | 48.92 | *p* < 0.001 | | 5 |
|  |  | PAML M7-M8 | 57.73 | *p* < 0.001 | | 5 |
|  |  |  |  |  | |  |
| BTR | B30.1 291 to 393 | PAML M1-M2 | 0.00 | NS^4^ | | 0 |
|  |  | PAML M7-M8 | 0.33 | NS^4^ | | 0 |
|  |  |  |  |  | |  |
| BTR | B30.1 430 to 557 | PAML M1-M2 | 7.02 | *p* < 0.05 | | 2 |
|  |  | PAML M7-M8 | 21.74 | *p* < 0.001 | | 2 |
|  |  |  |  |  | |  |
|  |  |  |  |  |  | |

^1^ for fragmented regions, numbers correspond with positions of first and last nucleotides in the alignment

^2^ The models M1a, M2a, M7 and M8 were employed, using either the program PAML or program PARRIS.

^3^ ND: sites are not determent under this model

^4^ NS: not significant.
