# Supplementary material for: Origin and Evolution of TRIM Proteins: New Insights from the Complete TRIM Repertoire of Zebrafish and Pufferfish
Source: PLoS One. 2011 Jul 15;6(7):e22022. doi: 10.1371/journal.pone.0022022 (PMC3137616; doi:10.1371/journal.pone.0022022)
Supplement: Figure S7 — A table showing the results of the GARD program for recombination between zebrafish trim35 and btr B30.2 domains. (DOC) [file pone.0022022.s007.doc]

FigureS7 GARD results

| domain | site nr |  | c-AIC | ∆ c-AIC ^2^ | Position^3^ | lnL |
| --- | --- | --- | --- | --- | --- | --- |
|  | in search ^1^ | BP |  |  |  |  |
| TRIM35 B30.2 | 1 |  | - | - | - | -12856 |
|  | 20 |  | - | - | - | -15774.2 |
| TRIM39 B30.2 | 1 | 0 | 18281.7 | - | - | -9082,15 |
|  |  | 1 | 18188.9 | 92.7883 | 288 |  |
|  |  | 2 | 18092.8 | 96.0918 | 262, 399 |  |
|  |  | 3 | 18092.8 | 0 | 262, 399 |  |
|  | 2 | 0 | 18281.7 | - | - | -9082,15 |
|  |  | 1 | 18188.9 | 92.7883 | 288 |  |
|  |  | 2 | 18092.8 | 96.0918 | 271, 427 |  |
|  |  | 3 | 18092.8 | 0 | 271, 427 |  |
|  | 3 | 0 | 18281.7 | - | - | -9082,15 |
|  |  | 1 | 18188.9 | 92.7883 | 288 |  |
|  |  | 2 | 18092.8 | 96.0918 | 269, 405 |  |
|  |  | 3 | 18092.8 | 0 | 269, 405 |  |
|  | 4 | 0 | 18281.7 | - | - | -9082,15 |
|  |  | 1 | 18188.9 | 92.7883 | 405 |  |
|  |  | 2 | 18092.8 | 96.0918 | 262, 405 |  |
|  |  | 3 | 18092.8 | 0 | 262, 405 |  |
|  | 5 | 0 | 18281.7 | - | - | -9082,15 |
|  |  | 1 | 18188.9 | 92.7883 | 405 |  |
|  |  | 2 | 18092.8 | 96.0918 | 262, 405 |  |
|  |  | 3 | 18092.8 | 0 | 262, 405 |  |
|  | 6 | 0 | 18281.7 | - | - | -9082,15 |
|  |  | 1 | 18188.9 | 92.7883 | 405 |  |
|  |  | 2 | 18092.8 | 96.0918 | 262, 405 |  |
|  |  | 3 | 18092.8 | 0 | 262, 405 |  |
|  | 7 | 0 | 18281.7 | - | - | -9082,15 |
|  |  | 1 | 18188.9 | 92.7883 | 405 |  |
|  |  | 2 | 18092.8 | 96.0918 | 262, 405 |  |
|  |  | 3 | 18092.8 | 0 | 262, 405 |  |
|  | 8 | 0 | 18281.7 | - | - | -9082,15 |
|  |  | 1 | 18188.9 | 92.7883 | 405 |  |
|  |  | 2 | 18092.8 | 96.0918 | 262, 405 |  |
|  |  | 3 | 18092.8 | 0 | 262, 405 |  |
|  | 9 | 0 | 18281.7 | - | - | -9082,15 |
|  |  | 1 | 18188.9 | 92.7883 | 405 |  |
|  |  | 2 | 18092.8 | 96.0918 | 271, 427 |  |
|  |  | 3 | 18092.8 | 0 | 271, 427 |  |
|  | 10 | 0 | 18281.7 | - | - | -9082,15 |
|  |  | 1 | 18188.9 | 92.7883 | 405 |  |
|  |  | 2 | 18092.8 | 96.0918 | 262, 405 |  |
|  |  | 3 | 18092.8 | 0 | 262, 405 |  |
|  | 11 | 0 | 18281.7 | - | - | -9082,15 |
|  |  | 1 | 18188.9 | 92.7883 | 405 |  |
|  |  | 2 | 18092.8 | 96.0918 | 262, 405 |  |
|  |  | 3 | 18092.8 | 0 | 262, 405 |  |
|  | 12 | 0 | 18281.7 | - | - | -9082,15 |
|  |  | 1 | 18188.9 | 92.7883 | 405 |  |
|  |  | 2 | 18092.8 | 96.0918 | 262, 405 |  |
|  |  | 3 | 18092.8 | 0 | 262, 405 |  |

^1^ For TRIM39, the GARD programmed was runned 12 times (for 20 sites)

^2^ The c-AIC, Akaike`s information criterium indicates which model fits the data best,

^3^ The positions of detected breakage points correspond with the nucleotide position in the alignment that we used for the GARD analysis. These positions are indicated on the B30.2 alignment, in figure 8.
